# Automating Chemical Reasoning in High‐Throughput Phase Identification With a Probabilistic, LLM‐Guided Framework

**DOI:** 10.1002/advs.76450

**Published:** 2026-08-03

**Authors:** Olympia Dartsi, Lauren N. Walters, Amalie E. Trewartha, Steven B. Torrisi, Amanda Volk, Liam Joyce, Gerbrand Ceder, Anubhav Jain

**Affiliations:** ^1^ Energy Technologies Area Lawrence Berkeley National Laboratory Berkeley USA; ^2^ Bakar Institute of Digital Materials for the Planet University of California Berkeley USA; ^3^ Materials Sciences Division Lawrence Berkeley National Laboratory Berkeley USA; ^4^ Energy & Materials Division Toyota Research Institute Los Altos USA; ^5^ Department of Materials Science & Engineering University of California Berkeley USA

## Abstract

Autonomous laboratories increasingly enable materials synthesis at scale, but traditional high‐throughput characterization workflows remain limited by the need for expert chemical intuition to distinguish plausible interpretations from formally good but chemically incorrect fits. We present an automated interpretation framework that combines probabilistic inference with automated chemical reasoning for phase identification from powder x‐ray diffraction (PXRD). The framework evaluates multiple candidate interpretations using diffraction pattern‐based metrics. It then refines these likelihoods using chemically‐informed priors derived from composition balance and a large language model (LLM)–based plausibility estimate with human‐readable justification and also produces a trustworthiness score. In a blinded multi‐project benchmark, the framework's top‐ranked interpretation was selected over the lowest‐$R_{\mathrm{wp}}$ baseline in 93% of cases where evaluators expressed a clear preference (95% CI: [78%, 98%], n=30). Trust decisions made by the framework aligned with expert judgment in approximately 75%–80% of cases. In a second evaluation, the framework systematically identified cases where lowest‐$R_{\mathrm{wp}}$ interpretations were chemically implausible and surfaced credible alternatives to historically ambiguous samples. By reframing phase identification as a problem of probabilistic reasoning and trust‐aware decision making, this work demonstrates how chemical intuition can be automated and scaled.

## Graphical abstract

Autonomous laboratories can now synthesize materials faster than experts can interpret the resulting diffraction data. A probabilistic framework combines refinement‐fit metrics with large language model‐derived chemical reasoning to rank competing phase interpretations and flag those unsuitable for autonomous use. Tests demonstrate that expert reviewers prefer its top‐ranked interpretation over the conventional lowest‐Rwp baseline.

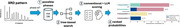

## 1 Introduction

Automated materials discovery has progressed to the point where synthesis, processing, and data acquisition can be executed at scale with minimal human intervention [1, 2, 3, 4, 5, 6, 7, 8, 9]. This acceleration is particularly consequential for sustainable‐materials research, where the pace of discovery directly affects progress on batteries, catalysts, photovoltaics, and replacements for critical or supply‐constrained elements. However, automated interpretation of characterization data remains an open question. In practice, the ability to extract chemically meaningful conclusions from experimental measurements remains tightly coupled to expert judgment, limiting scalability and reliability in high‐throughput campaigns. As a result, trustworthy characterization has emerged as a primary bottleneck to closed‐loop autonomous discovery [10].

Phase identification exemplifies this challenge. Determining which phases are present in a sample is essential for confirming reaction outcomes, identifying impurities, and guiding subsequent synthesis decisions. Powder x‐ray diffraction (PXRD) is among the most widely used tools for this purpose, yet experimental patterns frequently arise from complex phase mixtures, incomplete reactions, or structurally similar polymorphs. In such settings, multiple competing interpretations often explain the diffraction pattern nearly equally well, and small numerical differences in refinement metrics may not reflect meaningful chemical distinctions. When humans are confronted with such ambiguity, they implicitly or explicitly use their intuition and experience as cognitive bias to select one possible outcome over others. While PXRD analysis does not inherently produce a single answer, traditional workflows often collapse this ambiguity into a single preferred interpretation.

A range of computational approaches has sought to automate aspects of phase identification [11, 12]. Probabilistic deep‐learning models have demonstrated improved performance on multiphase diffraction patterns [13, 14], while Bayesian approaches have been used to formalize phase identification as a model selection problem, deriving posterior probabilities for candidate phases from physically motivated priors on peak shifts and intensity deviation [15, 16, 17]. In parallel, unsupervised phase‐mapping methods such as AgileFD have enabled automated decomposition of large high‐throughput diffraction datasets into constituent phase patterns, demonstrating the scalability of algorithmic XRD interpretation [18]. Despite these advances, existing approaches rely primarily on numerical similarity between experimental and simulated patterns [9, 13, 19, 20, 21]. As a result, they often fail to distinguish chemically distinct but crystallographically similar phases, such as closely related polymorphs, and may be more sensitive to experimental artifacts that introduce peak shifts, background variations, or noise. Analogous limitations have been observed in other characterization modalities, including x‐ray absorption near edge structure (XANES), where models operating directly on spectral features exhibit pronounced sensitivity to experimental variations [22].

These limitations have motivated increasing interest in incorporating human expertise into automated workflows. Human‐in‐the‐loop strategies enable domain knowledge to be injected into Bayesian phase mapping [23], while Bayesian algorithm execution encodes human expertise into high‐level scientific rules to guide experimental acquisition and decision‐making [24]. Collectively, these approaches demonstrate that effective automation in materials characterization depends on chemical intuition and historical knowledge beyond what is captured by purely numerical criteria [25]. Until recently, however, no practical mechanism existed to encode this intuition programmatically in a scalable and reproducible manner.

This work is enabled by a recent and fundamental shift: large language models (LLMs) now provide a mechanism to potentially automate messy, qualitative chemical reasoning in a rapid, reproducible, and computationally accessible form. LLMs provide an opportunity to assess whether a phase can reasonably form under given experimental conditions, or whether a proposed phase mixture is chemically coherent, without hand‐authoring exhaustive rule sets. This capability opens a new path for integrating chemical intuition directly into automated characterization pipelines.

Here, we introduce an automated interpretation framework that leverages this capability by combining probabilistic inference with scalable chemical reasoning for phase identification from powder x‐ray diffraction. The framework evaluates multiple candidate interpretations using diffraction‐based metrics, including the weighted‐profile R‐factor ($R_{\mathrm{wp}}$) and a peak‐matching score, and refines these likelihoods using chemically informed priors. These priors include a composition‐balance score that enforces elemental consistency and an LLM‐derived plausibility estimate that encodes chemistry‐aware priors aligned with expert judgement in the regimes evaluated. The plausibility estimate provides both numerical scores as well as human‐readable rationales for phase plausibility.

The framework separates probabilistic ranking of possible solutions from decision‐making. In addition to assigning posterior probabilities to candidate interpretations and individual phases, it computes an independent trustworthiness score that assesses whether an interpretation is sufficiently supported to be acted upon autonomously or should be deferred for human review. This distinction is essential in settings where large numbers of samples must be evaluated with limited human oversight, such as high‐throughput campaigns, where many cases are intrinsically ambiguous and overconfident automation can propagate errors downstream.

We demonstrate the utility of this approach through expert‐blinded benchmarks, large‐scale high‐throughput evaluations, and targeted case studies involving ambiguous phase mixtures and competing structural hypotheses. More broadly, this work illustrates how LLMs can serve as scalable surrogates for chemical intuition in automated interpretation tasks.

## 2 Methods

### 2.1 Architecture of the Automated Interpretation Framework

The automated interpretation framework (AIF) reframes phase identification as a problem of probabilistic interpretation rather than single‐solution optimization. Instead of selecting a single “best‐fit” refinement‐based on the lowest weighted‐profile residual ($R_{\mathrm{wp}}$), AIF evaluates and ranks multiple competing phase hypotheses according to their posterior probabilities. This shift enables uncertainty, plausibility, and chemical reasoning to be treated as first‐class quantities in high‐throughput characterization workflows.

**FIGURE 1:**
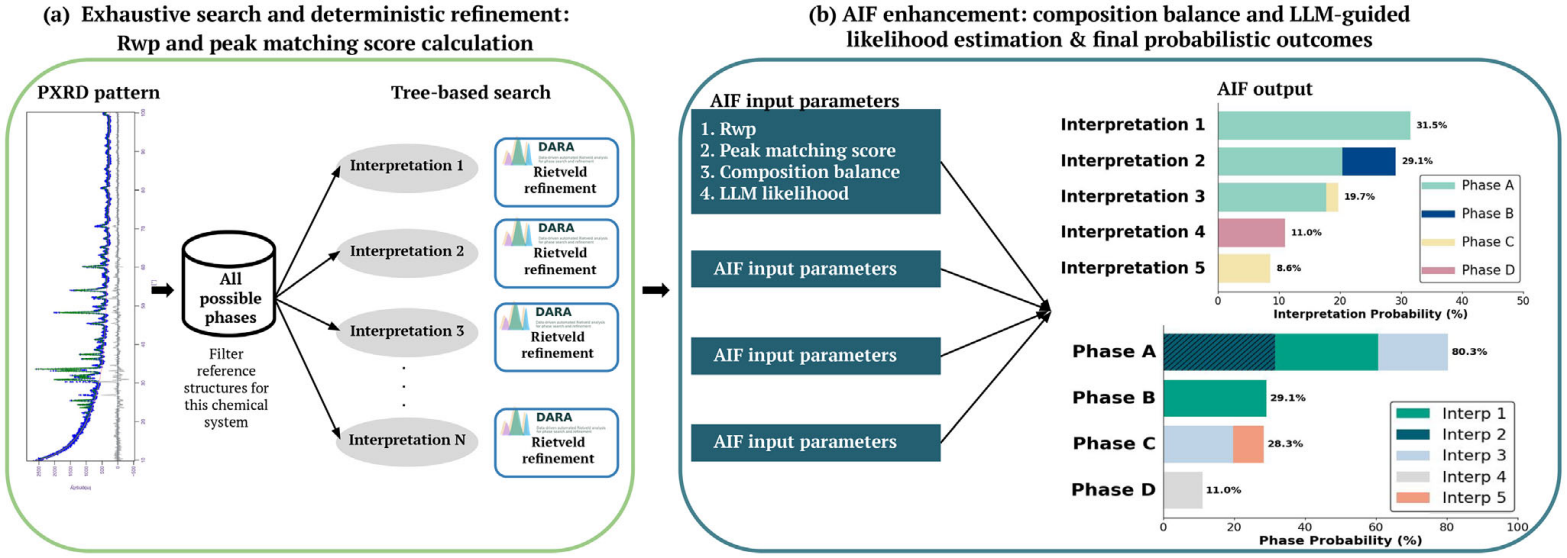
Overview of AIF for phase identification from PXRD. (a) The workflow begins with the acquisition of a PXRD pattern (left), which is used to enumerate candidate phase combinations. These are evaluated through a tree‐based search and Rietveld refinement pipeline, representing the traditional exhaustive fitting approach. (b) For each candidate interpretation, AIF computes chemistry‐aware priors, a composition‐balance score and an LLM‐derived plausibility likelihood. These metrics are combined in a Bayesian scoring model to rank interpretations and to infer marginal phase probabilities, yielding interpretation‐level and phase‐level probability outputs.

Figure 1 provides an overview of the framework. As shown in Figure 1A, the workflow begins with a conventional PXRD pattern, which is analyzed using a standard refinement pipeline. Reference crystallographic information files (CIFs) are obtained from user‐specified crystallographic databases, and diffraction data (diffractograms) are obtained by the A‐Lab autonomous laboratory from inorganic bulk solid‐state synthesis at LBNL [1]. To constrain the hypothesis space to chemically relevant candidates, the structural database is filtered to retain only phases whose constituent elements form a subset of those specified in the synthesis recipe.

From this filtered pool, AIF constructs candidate interpretations as subsets of possible phase combinations whose constituent elements are consistent with those present in the precursors. The exploration of this hypothesis space is carried out using the tree‐based search and automated refinement machinery provided by the Dara framework [26]. Each node in the search tree corresponds to a candidate interpretation, a specific combination of one or more crystalline phases, and child nodes are generated by incrementally adding phases. Because exhaustive enumeration of all phase combinations is combinatorial, the search is prioritized and pruned using a fast peak‐matching heuristic to down‐select promising candidates prior to full refinement. Retained candidates are then evaluated using Rietveld refinement [27, 28, 29, 30], yielding quantitative fit metrics such as $R_{\mathrm{wp}}$ and a peak‐matching score that assess agreement between simulated and observed diffraction patterns (Section S11.2). The search terminates when a maximum phase count is reached or when additional phases no longer improve the fit, producing a set of well‐fitting candidate interpretations.

The number of retained interpretations varies with system complexity; in practice, this procedure yields between 4 and 15 candidate interpretations per diffraction pattern, with most samples producing approximately 4–8 viable hypotheses. Each candidate interpretation corresponds to a mixture of one or more crystalline phases, with refined phase fractions obtained through Rietveld refinement analysis (Section S9).

As illustrated in Figure 1b, AIF augments these refinement‐based fits with chemistry‐aware priors that introduce chemical knowledge in a form compatible with high‐throughput automation. In the current implementation, two complementary sources of chemical information are used. A composition‐balance score evaluates whether the elemental composition implied by an interpretation is consistent with the target stoichiometry, while explicitly allowing for exchange with the environment, e.g., loss of volatile species such as $O_{2}$, ${\mathrm{CO}}_{2}$, or $H_{2}O$ (Section S11.1). In parallel, an LLM‐derived plausibility score estimates whether individual phases and phase combinations are chemically reasonable under the reported synthesis conditions using a structured few‐shot prompt (Section S10).

Finally, as shown in Figure 1b, refinement‐based figures of merit ($R_{\mathrm{wp}}$ and peak‐matching score) and chemistry‐informed priors (composition balance and LLM plausibility) are combined in a unified Bayesian formulation to compute posterior probabilities. In addition to a quantitative plausibility score, the LLM returns a structured natural‐language explanation that enumerates the chemical factors influencing each phase‐level likelihood (e.g., redox compatibility, polymorph stability, precursor decomposition behavior), enabling direct inspection of the reasoning behind each assessment. Given all the component scores, the framework outputs both interpretation‐level posterior rankings and phase‐level marginal probabilities, highlighting not only the most likely overall explanation for a diffraction pattern, but also the phases most consistently supported across competing hypotheses.

The chemical priors introduced here are intentionally minimal and interpretable, providing chemistry‐informed constraints rather than an exhaustive encoding of chemical knowledge. The framework is modular by design and can, in principle, incorporate additional sources of information in future implementations, including computational thermodynamic or kinetic models (e.g., formation energies relative to convex hulls), constraints or penalties on refined Rietveld parameters (such as physically implausible strain or microstructural values), or prior knowledge about whether candidate phases are experimentally established or hypothetical. While the present work focuses exclusively on PXRD, the underlying architecture—probabilistic hypothesis evaluation augmented by domain‐informed priors—is formulation‐agnostic and naturally supports extensions to joint probabilistic inference across multiple characterization modalities (e.g., combined PXRD and SEM–EDS likelihoods). In this sense, the framework serves as a conceptual blueprint for scalable, trust‐aware automated interpretation rather than a modality‐specific solution.

### 2.2 Probabilistic Bayesian Formulation

The Bayesian formulation of AIF quantifies the probability of candidate interpretations and constituent phases given an observed diffraction pattern. The framework decomposes the inference task into three complementary components: interpretation‐level posterior probabilities, phase‐level posterior probabilities, and an assessment of overall interpretation trustworthiness.

At the interpretation level, we compute the posterior probability $P(I_{n}|D)$ for each candidate interpretation $I_{n}$, where D denotes the observed powder x‐ray diffraction pattern (diffractogram). An interpretation is defined as a combination of crystalline phases $\left\{f_{i}\right\}$ with associated refined phase fractions $\left\{x_{i}\right\}$. This quantity captures confidence in the complete PXRD interpretation by jointly accounting for the identities and relative abundances of all phases contributing to the observed diffraction pattern.

At the phase level, we compute the marginal posterior probability $P(f_{i}|D)$ for each phase $f_{i}$, representing the likelihood that a given phase is present in the measured diffraction pattern. These probabilities are obtained by aggregating evidence across all candidate interpretations containing $f_{i}$.

In addition to Bayesian probabilities, AIF evaluates the reliability of each interpretation using diagnostics derived from diffraction data quality, refinement behavior, and chemically informed plausibility metrics. These diagnostics are combined to produce a continuous trustworthiness score that identifies interpretations with elevated uncertainty or limited evidentiary support.

These components are described in greater detail in the subsequent subsections.

#### 2.2.1 Interpretation‐level Posterior Probability

The posterior probability of a candidate interpretation $I_{n}$ given an observed diffraction pattern D is defined according to Bayes' theorem as

(1) $$P\left(I_{n}|D\right)=\frac{P\left(D|I_{n}\right)\;P\left(I_{n}\right)}{P(D)},$$

where an interpretation $I_{n}$ is defined as a combination of one or more crystalline phases $\left\{f_{i}\right\}$, each associated with a refined phase fraction $\left\{x_{i}\right\}$, whose combined diffraction pattern accounts for the observed diffraction pattern. Since the measured diffraction pattern D is fixed, the evidence term P(D) is identical across all candidate interpretations and cancels when comparing them.

The likelihood term $P(D|I_{n})$ quantifies the degree of agreement between the diffraction pattern simulated from interpretation $I_{n}$ and the observed diffraction pattern. In the present implementation, this likelihood is computed as a weighted average of normalized diffraction pattern‐based evaluation metrics,

(2) $$P\left(D|I_{n}\right)=\frac{\sum_{k}w_{k}\;m_{k}^{\left(\mathrm{spec}\right)}}{\sum_{k}w_{k}},$$

where $m_{k}^{\left(\mathrm{spec}\right)}$ denotes an individual spectral metric and $w_{k}$ its associated weight. The spectral metrics used include the weighted profile residual $R_{\mathrm{wp}}$ [31], which measures the overall numerical quality of the Rietveld refinement, and a composite peak‐matching score, introduced in Dara [26], that quantifies the correspondence between simulated and observed diffraction peak positions and intensities. Details of the definition of the peak‐matching score, and its implementation are provided in Section S11.2.

The prior probability $P(I_{n})$ encodes the chemically informed plausibility of interpretation $I_{n}$ prior to considering diffraction evidence. It is expressed as a weighted combination of normalized interpretation‐level prior metrics

(3) $$P\left(I_{n}\right)=\frac{\sum_{\ell }w_{\ell }\;m_{\ell }^{\left(\mathrm{prior}\right)}}{\sum_{\ell }w_{\ell }},$$

where each $m_{\ell }^{\left(\mathrm{prior}\right)}$ reflects a distinct aspect of chemical realism. In this work, the prior incorporates an LLM‐derived plausibility score, which evaluates the feasibility of the proposed phases and their coexistence under the synthesis conditions, and a composition‐balance score, which quantifies consistency with elemental conservation and expected mass balance given the precursor set. Details of the composition‐balance score calculation are given in the Section S11.1.

All scoring terms are scaled to a common [0,1] scale before combination. The composition‐balance score (Section S11.1), the LLM‐derived plausibility score (elicited by prompt design; Section S10), and the Dara‐derived peak‐matching score (Section S11.2) are each defined on this bounded interval. By contrast, the weighted profile R‐factor $R_{\mathrm{wp}}$ from refinement is unbounded, so we map it to [0,1] using a clipped linear normalization: values of $R_{\mathrm{wp}}=0$ are mapped to 1, and values at or above a maximum cutoff of $R_{\mathrm{wp}}=60$ are mapped to 0, thereby limiting the influence of extreme outliers.

#### 2.2.2 Phase‐level Posterior Probability

The posterior probability that an individual phase $f_{i}$ is present in the sample is obtained by summing over all candidate interpretations $I_{n}$. Each interpretation corresponds to a specific combination of phases and associated phase fractions, with posterior probability $P(I_{n}|D)$ determined from the diffraction data.

The phase‐level posterior probability is given by

(4) $$P\left(f_{i}|D\right)=\sum_{I_{n}}P\left(I_{n}|D\right)\;P\left(f_{i}|I_{n}\right),$$

where $P(f_{i}|I_{n})$ is an indicator function that equals unity if phase $f_{i}$ is present in interpretation $I_{n}$ and zero otherwise. Under this definition, Equation (4) reduces to a sum of posterior probabilities over all interpretations containing phase $f_{i}$

(5) $$P\left(f_{i}|D\right)=\sum_{I_{n}∋f_{i}}P\left(I_{n}|D\right).$$

This formulation provides a quantitative measure of the marginal posterior probability of each phase across all candidate interpretations.

#### 2.2.3 Interpretation Trustworthiness Assessment

In addition to assigning Bayesian posterior probabilities to phases and complete interpretations, AIF quantifies the reliability of each candidate interpretation through a complementary trustworthiness score that augments the chemistry priors used in the posterior with additional data‐quality and refinement‐stability diagnostics. Whereas posterior probabilities provide a relative ranking among competing interpretations for a given sample, they do not indicate whether a particular interpretation is sufficiently reliable to act upon. The trustworthiness score addresses this limitation by evaluating diffraction data quality, refinement stability, and chemical plausibility on a per‐interpretation basis, thereby enabling the framework to distinguish interpretations that can be used directly in automated workflows from those that require human intervention.

For each interpretation $I_{n}$ and observed diffraction pattern D, AIF computes a trustworthiness score $T\left(I_{n}|D\right)\in \left[0,1\right]$ as one minus the average of a set of bounded diagnostic penalties:

(6) $$T\left(I_{n}|D\right)\;=\;1-\frac{1}{K}\sum_{k=1}^{K}p_{k}\left(I_{n},D\right),$$

where each penalty $p_{k}\left(I_{n},D\right)\in \left[0,1\right]$ is computed by first measuring the deviation of the corresponding diagnostic value from a predefined reference threshold associated with poor or unreliable behavior, and then mapping this deviation to a bounded penalty using a logistic function. Diagnostics that lie well within acceptable regimes yield penalties close to zero, while increasingly severe deviations into unfavorable regimes yield penalties approaching unity. The sharpness of this transition is controlled by diagnostic‐specific scale parameters, which are fixed across all experiments.

The trustworthiness score aggregates a small set of physically motivated diagnostics capturing diffractogram signal quality, refinement behavior, and chemically informed plausibility. Each diagnostic contributes a bounded penalty that is aggregated according to Equation (6), yielding a continuous trustworthiness score rather than a binary decision. Low trustworthiness values therefore indicate elevated uncertainty in the interpretation, without altering the Bayesian ranking among competing candidates.

Several alternative figures of merit and indicators of reliability were considered, including raw weighted profile R‐factor ($R_{\mathrm{wp}}$) values, raw chemistry prior scores, posterior probabilities, and interpretation entropy (e.g., whether many different interpretations were equally favored). However, none of these quantities alone provides a reliable measure of trustworthiness and many of them did not help in distinguishing trustworthy interpretations. Therefore, we adopt a composite trustworthiness score that integrates multiple orthogonal diagnostics, capturing complementary failure modes that are not robustly identified by any single metric.

Table 1 summarizes the diagnostic quantities and reference values used to parameterize the penalty functions contributing to the trustworthiness score.

**TABLE 1:** Diagnostic quantities, reference thresholds, and logistic scale parameters used in the trustworthiness penalty function (Equation 6). A global temperature of T=1.25 and uniform weights $w_{k}=1.0$ are applied to all diagnostics. Diagnostics are combined continuously rather than applied as independent binary flags.

Criterion	Reference threshold	Scale parameter
Low Signal	< 9000	2000.0
High Background	> 1200	300.0
Signal/Background ratio	< 15	3.0
LLM interpretation likelihood	≤ 0.4	0.10
Balance Score	< 0.6	0.10
Peak matching score	< 0.6	0.10
$R_{\mathrm{wp}}$	> 15	5.0

These diagnostics do not alter the Bayesian ranking of interpretations, but instead highlight cases where further examination is required.The full definitions of the underlying diagnostics and their normalization are provided in Section S11.3.

Each diagnostic contributes a graded penalty to Equation (6), with penalties increasing smoothly as diagnostic values deviate from their reference regimes and capped to prevent any single diagnostic from dominating the trustworthiness score. In this work, interpretations with a combined trustworthiness score below 0.6 are conservatively labeled as untrustworthy and treated uniformly as such in all subsequent analyses.

### 2.3 Evaluation Methodology

**FIGURE 2:**
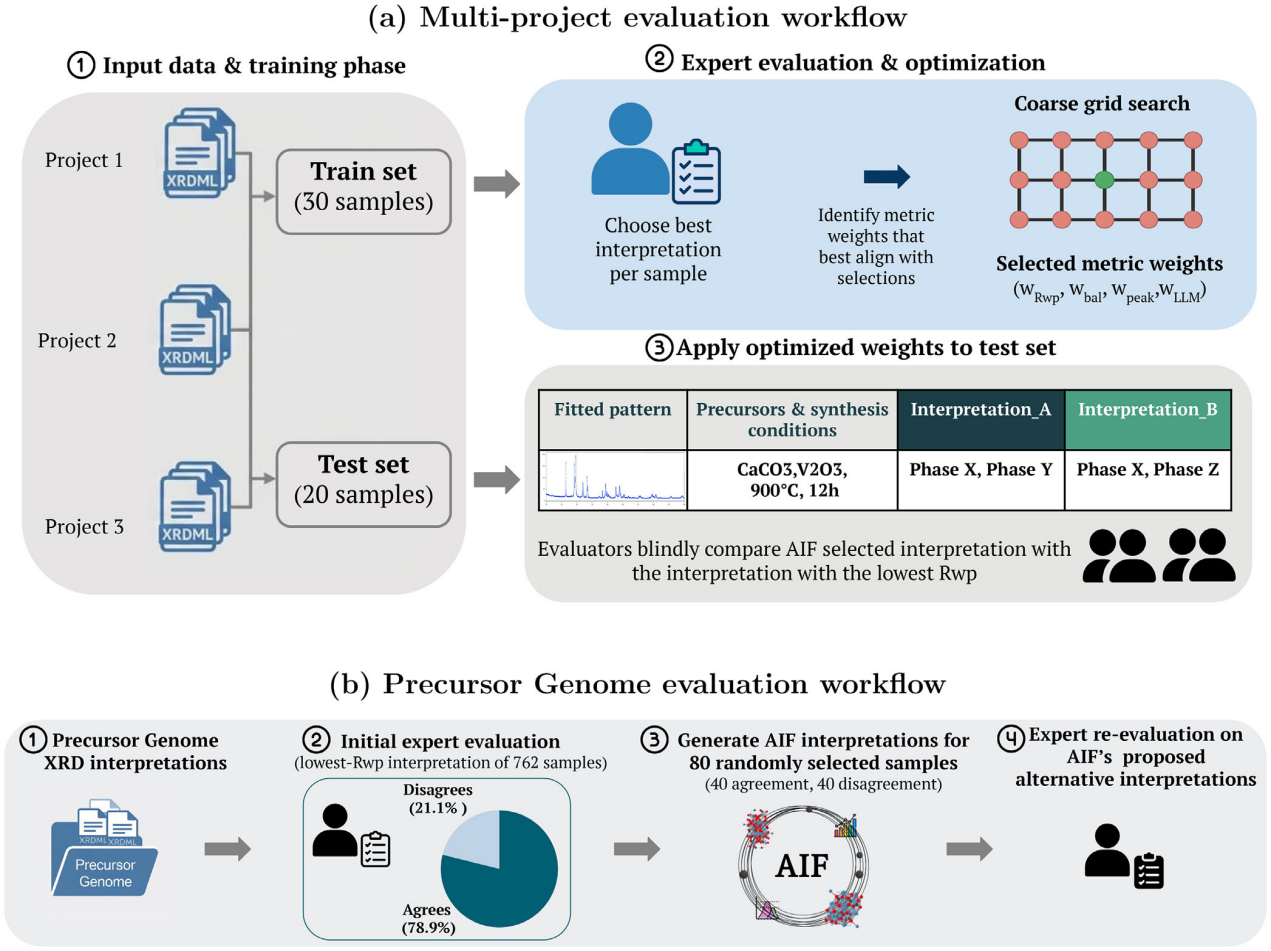
Evaluation workflows used in this work. (a) Multi‐project blinded expert comparison between AIF's top‐ranked interpretation and the lowest‐$R_{\mathrm{wp}}$ interpretation after calibration on 30 samples; 20 held‐out samples were used for evaluation by 4 chemists (80 total evaluations). (b) Precursor Genome re‐evaluation workflow: an expert first labeled 762 lowest‐$R_{\mathrm{wp}}$ interpretations as satisfactory/unsatisfactory, then re‐evaluated a balanced subset of 80 samples by comparing AIF's top‐ranked interpretation to the original lowest‐$R_{\mathrm{wp}}$ interpretation.

To validate the effectiveness of the AIF, we designed two complementary evaluations that probe expert agreement and expert revision (Figure 2). The first involves a blinded comparison of the AIF‐selected interpretation with the lowest‐$R_{\mathrm{wp}}$ selection, defined as the candidate yielding the minimum weighted profile R‐factor (best numerical fit) in Rietveld refinement, across multiple synthesis projects and evaluated by four independent chemists to assess decision‐level agreement under realistic experimental conditions. The second uses a controlled subset of the Precursor Genome dataset [32], which includes PXRD characterization of solid‐state syntheses of common precursor pairs, where an expert re‐evaluates cases where AIF proposes an interpretation different from the previously evaluated lowest‐$R_{\mathrm{wp}}$ interpretation, testing whether AIF can identify credible or improved alternatives in historically ambiguous cases. The structure model interpretation with lowest‐$R_{\mathrm{wp}}$ is used as a baseline to compare against AIF due to the $R_{\mathrm{wp}}$ being a common discrepancy value reported in the diffraction community to report refinement error [33], and to its frequent use as the primary figure of merit for ranking interpretations in other automated PXRD analysis tools [9, 19, 20, 21].

#### 2.3.1 Evaluation on Multi‐project Data

A multi‐project evaluation was designed to assess AIF performance across a broad and experimentally realistic range of synthesis conditions, chemistries, and diffraction complexities. To construct the evaluation workflow (Figure 2a), we aggregated diffraction characterization data from multiple synthesis efforts, including historical A‐Lab datasets, the TRI new‐compounds dataset, and the Precursor Genome dataset, yielding a total of over 100 candidate samples spanning diverse chemical systems and experimental conditions. The TRI new‐compounds dataset is a set of experiments primarily designed to probe the stability ranges of various oxidation states of transition metals such as V, Cr, Cu and Ag under different temperature and reducing gas conditions. The Precursor Genome [32] is a set of inorganic solid‐state synthesis reactions between pairs of common precursors, executed with the automated self‐driving laboratory, A‐Lab [1]. A collection of 46 different precursors common in battery material research and spanning 37 different elements were reacted. This precursor set spans the transition‐metal oxides, phosphates, and layered hosts that form the backbone of lithium‐ion and next‐generation battery chemistries, making it directly representative of the sustainable‐energy materials space that autonomous laboratories are increasingly tasked with exploring.

From this pool, samples were curated to remove redundant cases, including instances with identical or near‐identical synthesis conditions, highly similar phase interpretations, and cases where AIF and the lowest‐$R_{\mathrm{wp}}$ solution yielded the same preferred interpretation. This curation was performed to ensure that human evaluators were not repeatedly exposed to effectively equivalent data. The resulting dataset was then divided into a calibration subset used to tune model parameters and a held‐out test subset used exclusively for evaluation. A subset of 30 samples was used for calibration, and an additional 20 samples were reserved for independent evaluation by four chemists (80 total test evaluations).

For the training set, a single domain expert—who was not among the four chemists involved in the final evaluation—manually reviewed the complete set of candidate interpretations for each sample. This separation was intentionally enforced to avoid biasing the subsequent human evaluation and to prevent implicit alignment between the calibrated AIF weights and the preferences of the evaluators. For each sample, the expert selected the interpretation they judged to be the most physically and crystallographically plausible. These selections served as reference labels for calibrating the relative weights of AIF's data‐driven and chemically informed metrics.

As discussed in Section 2.2.1, AIF assigns a probability to each candidate interpretation using a weighted combination of four metrics: $R_{\mathrm{wp}}$, peak‐matching score, composition‐balance score, and LLM‐derived likelihood. To determine the relative importance of these metrics ($w_{k}$ in Equation 2), we performed a coarse multidimensional grid search over candidate weight combinations (Section S12.1.1). The search explored joint variations of all four metric weights at low resolution, and selected the combination that maximized agreement with expert‐selected interpretations. This calibration required only a small subset of labeled samples and was intended to capture relative metric importance rather than precise optimal values. The resulting optimized weights are:

$$w=\{\;w_{R_{\mathrm{wp}}}=1.0,\;w_{\mathrm{bal}}=0.7,\;w_{\mathrm{peak}}=0.5,\;w_{\mathrm{LLM}}=0.5\;\}.$$

These values reflect a hierarchy that aligns with common practice. The weighted profile residual remains pivotal ($w_{R_{\mathrm{wp}}}=1.0$), indicating that a low $R_{\mathrm{wp}}$ is still the strongest global indicator of refinement quality. The composition‐balance score is also heavily weighted ($w_{\mathrm{bal}}=0.7$), emphasizing that elemental completeness and realistic stoichiometry are nearly as critical as the fit itself. Peak‐matching agreement contributes with moderate weight ($w_{\mathrm{peak}}=0.5$), adding sensitivity to local peak positions and intensities not fully captured by $R_{\mathrm{wp}}$. The LLM‐derived phase likelihood ($w_{\mathrm{LLM}}=0.5$) provides a chemistry‐aware prior that favors structurally plausible phases under the synthesis conditions; this weight is below $w_{R_{\mathrm{wp}}}$ but equal to the peak‐matching contribution, sized so that the LLM is decisive in chemically ambiguous regimes while remaining subordinate to overall refinement quality. Although calibration relied on a single expert's labels, to assess whether this introduced systematic bias, we compared AIF's top‐ranked interpretation against the majority preference of the four independent evaluators on the held‐out test set. Among the 10 samples where a clear majority preference existed, AIF matched the majority in all 10 cases (100%), suggesting that the calibration expert's judgment was consistent with the broader evaluator pool. To further assess robustness, ±0.1 perturbations on each weight were tested independently on the held‐out test set; in 19 out of 20 samples the top‐ranked interpretation was unchanged, and the one sample showing sensitivity switched between two interpretations with posterior probabilities differing by less than 1%. Further details on the grid search, score distribution, and perturbation analysis are provided in Section S12.1.1.

The calibrated AIF model was then applied to the test set that was not used during weight calibration. Cases in which AIF and the lowest‐$R_{\mathrm{wp}}$ interpretation coincided were excluded, as presenting identical interpretations would not allow a meaningful comparative evaluation. These samples span 15 distinct oxide chemistries, including several chemical and structural families, including vanadium oxides, mixed‐metal phosphates, tungstates, silver–manganese oxides, tantalate–lead oxides and tunnel manganese oxides. A full summary of chemistries and synthesis conditions is provided in Table S1. Each sample was evaluated independently by four chemists in a fully blinded manner. For every case, reviewers were presented with two candidate interpretations: the AIF top‐ranked interpretation, and the interpretation corresponding to the lowest refined $R_{\mathrm{wp}}$ value with no indication of which method suggested which interpretation.

Chemists assigned a numerical score on a [0,0.5,1] scale to each interpretation and then indicated whether one interpretation was preferred, whether both were acceptable, or whether neither adequately explained the diffraction pattern. When assigning lower scores, reviewers were asked to indicate the reason, including missing peaks, extra peaks, incorrect relative intensities, composition‐balance inconsistencies, or omission of expected phases.

Using multiple chemists allows us to explicitly characterize the variability and subjectivity inherent in real‐world phase interpretation, rather than implicitly assuming a single authoritative ground truth. As shown in Figure S3, substantial inter‐annotator disagreement persists even among experienced practitioners, highlighting the limitations of deterministic decision rules based on a single metric such as $R_{\mathrm{wp}}$. This observed variability motivates a probabilistic treatment of phase interpretation, in which competing hypotheses and their associated uncertainty can be represented explicitly.

#### 2.3.2 Evaluation on Precursor Genome data

To further assess AIF's ability to propose chemically meaningful alternative interpretations, we used a large subset of the Genome diffraction characterization dataset, in which each diffraction pattern had previously been reviewed by a chemist who evaluated the interpretation selected via the lowest‐$R_{\mathrm{wp}}$ refinement, using chemical judgment (workflow summarized in Figure 2b).

For this evaluation, we used a curated subset of the 762 total Genome diffraction patterns. The refinement fit for each diffraction pattern used herein was generated automatically from a structure model identified by a search‐tree algorithm [26], and selected utilizing the lowest‐$R_{\mathrm{wp}}$ ranking method. Selected interpretations were subsequently inspected manually by an expert.

As shown in Figure 2b, 78.9% of these lowest‐$R_{\mathrm{wp}}$ interpretations were judged acceptable, while 21.1% were deemed unsatisfactory—highlighting cases in which relying solely on the lowest‐$R_{\mathrm{wp}}$ can lead to chemically implausible or incomplete interpretations.

To compare AIF's top‐ranked interpretations with these manual annotation labels, we selected a balanced subset of 80 samples, comprising 40 cases in which the expert had previously judged the automatically ranked lowest‐$R_{\mathrm{wp}}$ interpretation satisfactory and 40 cases judged unsatisfactory. For each selected sample, we generated the full AIF candidate set and extracted the AIF top‐ranked interpretation. The same expert then re‐evaluated each AIF result and compared it directly against the original, automated lowest‐$R_{\mathrm{wp}}$ interpretation, judging whether AIF's solution was superior, equivalent, or inferior. The results of this targeted study are presented in Section 3.2.

## 3 Results

### 3.1 Evaluation on an Independent Expert‐Reviewed Multiproject Test Set

As outlined in the Methods (Section 2.3.1), an independent test set of 20 synthesized samples was reviewed in a blinded manner by four chemists. The detailed synthesis conditions, phases present per interpretations and interpretation preference per chemists are provided in Table S2. Here, we report the outcomes of these evaluations.

For each synthesized sample, chemists were presented with two candidate interpretations: (i) the interpretation ranked highest by AIF, and (ii) the interpretation corresponding to the refinement with the lowest $R_{\mathrm{wp}}$. Chemists were blinded to which interpretation was proposed by AIF and which was the lowest‐$R_{\mathrm{wp}}$ solution. For each pair, chemists were asked to indicate their preference by selecting one of four options: AIF preferred, lowest‐$R_{\mathrm{wp}}$ preferred, both acceptable, or neither acceptable. This design allows us to distinguish cases where AIF clearly improves interpretability from those that remain ambiguous or unsatisfactory under both approaches.

**FIGURE 3:**
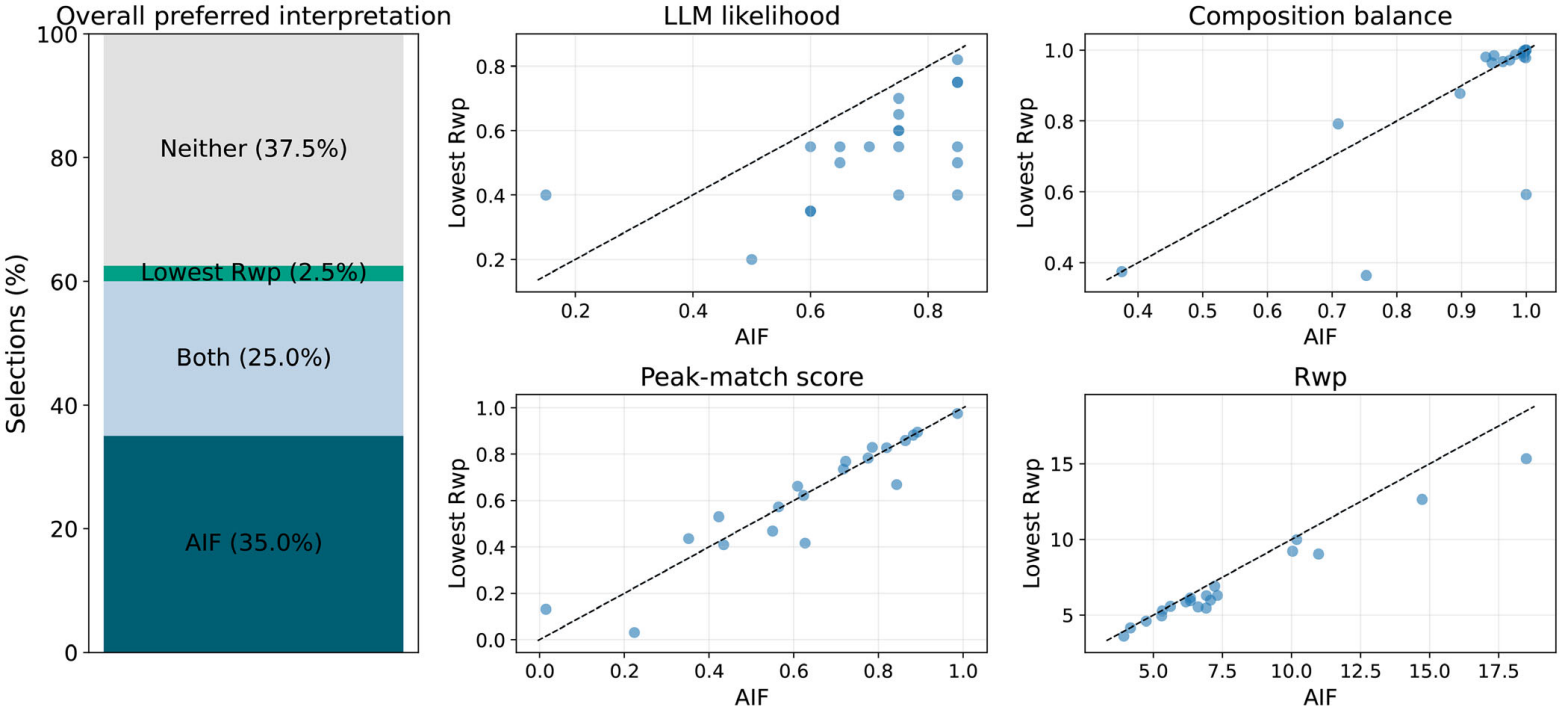
Expert evaluation results on the independent 20‐sample benchmark dataset. The stacked bar chart (left) summarizes the overall distribution of chemist preferences across all samples. Reviewers could select the AIF interpretation, the lowest‐$R_{\mathrm{wp}}$ interpretation, both interpretations as acceptable, or neither. The panels on the right compare the values of the four underlying metrics (LLM likelihood, composition balance, peak‐matching score, and $R_{\mathrm{wp}}$) between the AIF‐selected interpretation and the lowest‐$R_{\mathrm{wp}}$ interpretation for each sample.

The completed evaluations were aggregated to quantify per‐chemist preferences across the dataset, summarized in Figure 3.

Overall, chemists preferred the AIF top‐ranked interpretation in 35% of cases (28/80; Wilson 95% CI: [25%, 47%]), compared to only 2.5% for the lowest‐$R_{\mathrm{wp}}$ refinement. Furthermore, when considering only cases in which chemists expressed a clear preference for one interpretation over the other, the AIF top‐ranked interpretation was selected in 93% of evaluations (28/30; Wilson 95% CI: [78%, 98%]). The remaining cases were split between both interpretations deemed acceptable (25%) and neither interpretation deemed acceptable (37.5%). The large “neither acceptable” category is noteworthy: it indicates that a substantial fraction of real experimental diffraction patterns remain difficult to interpret, highlighting the importance of a trustworthiness score that can represent uncertainty in ambiguous cases.

To understand why chemists preferred one interpretation over the other, we next compare the underlying metric values assigned to the AIF‐selected interpretation and the lowest‐$R_{\mathrm{wp}}$ interpretation. The scatter plots in the right panel of Figure 3 compare the raw unweighted values of the four underlying metrics (LLM likelihood, composition balance, peak‐matching score, and $R_{\mathrm{wp}}$) between the AIF‐selected interpretation and the interpretation with the lowest‐$R_{\mathrm{wp}}$. Each point corresponds to a single sample, with the diagonal indicating parity between the two interpretations. These comparisons isolate how individual metrics differ between the two candidates, independent of the final posterior weighting.

The AIF‐selected interpretation has a higher LLM likelihood than the lowest‐$R_{\mathrm{wp}}$ interpretation in 95% of samples, with 20% of the cases showing a difference of |Δ|>0.3. The composition balance score is nearly identical between the two interpretations in almost all cases, although two samples exhibit large differences (Δ≈0.4) in favor of the AIF, corresponding to important corrections of chemically implausible stoichiometries. Peak‐matching scores are also close to parity (75% with difference less than 0.1), and no sample shows differences greater than 0.3. As expected, the lowest‐$R_{\mathrm{wp}}$ refinement achieves lower $R_{\mathrm{wp}}$ values by construction. While most $R_{\mathrm{wp}}$ differences fall between 0.3 and 1.1, only three samples exceed a difference of 1.5, and just one sample shows a very large deviation (|Δ|=3.17). These isolated cases demonstrate that $R_{\mathrm{wp}}$ varies substantially between refinements, but such large deviations are uncommon and do not necessarily correspond to improvements in the chemically driven metrics.

The left panel of Figure S3 shows per‐chemist preferences; all reviewers favored AIF's interpretations more frequently than the lowest‐$R_{\mathrm{wp}}$ solutions. Across all 20 samples, only a single case was identified in which at least two chemists selected the lowest‐$R_{\mathrm{wp}}$ solution as the more credible interpretation. Although chemists are anonymized in the figures, their backgrounds provide context for the observed variability: Reviewer A has the most extensive experience in solid‐state synthesis and diffraction, Reviewer B has substantial prior refinement experience, and Reviewers C and D have primarily theoretical backgrounds with moderate exposure to diffraction refinement. These experience gradients are reflected in the inter‐chemist agreement matrix shown in the left panel of Figure S3. Summed strict pairwise agreement values are A: 185%, B: 160%, C: 150%, and D: 135%. In this strict definition, two chemists are considered to agree only when they select exactly the same category (AIF, Lowest $R_{\mathrm{wp}}$, Both, or Neither). Pairwise agreement levels span 35% –70% across reviewer pairs, indicating substantial subjectivity even among trained chemists. A less strict “partial agreement” analysis—where categories such as *Both* are considered compatible with either AIF or Lowest $R_{\mathrm{wp}}$—yields pairwise agreement levels of 65% –85% (Figure S4). This level of disagreement is consistent with prior studies of human labeling variability in diffraction analysis, such as Hattrick‐Simpers et al. (NIST, 2021)[34], who likewise reported substantial expert divergence, including dozens of composition–temperature points with non‐zero labeling entropy and one annotator whose labels differed qualitatively from the rest. This variability underscores the need to preserve multiple possible interpretations of a characterization result through a probabilistic framework such as AIF.

Details of the refinement results and synthesis conditions for this sample are provided in Section S12.1.3.

#### 3.1.1 Trust Alignment Between AIF and Chemists

**FIGURE 4:**
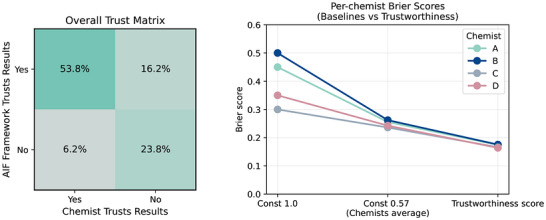
The matrix in the left panel shows how often AIF and the chemists made the same trust decision (both trust or both withhold trust) versus cases of asymmetric trust. The panel on the right reports the Brier scores comparing AIF's trustworthiness measure against constant baselines.

We next examined whether AIF assigns high confidence to the same interpretations trusted by the chemist annotators. The left panel of Figure 4 shows the overall trust alignment. In 53.8% of samples, AIF and the chemists jointly trusted the same interpretation. An additional 23.8% of samples showed mutual non‐trust, meaning that in a total of 77.6% of cases both AIF and the chemists agreed on whether an interpretation should be trusted. The remaining cases reflect asymmetric trust: chemists did not trust an interpretation that AIF scored as trustworthy in 16.2% of samples, while the reverse occurred in only 6.2%. Per‐chemist information is provided in the Supporting Information, including trust matrices (Figure S6) and ROC curves assessing discriminative ability across chemists and Brier score‐based calibration comparisons against baseline models (Section S12.3).

To assess whether the trustworthiness score behaves like a meaningful probability, we computed Brier scores [35] for each chemist and compared them against two baselines. The Brier score measures the mean squared error between predicted probabilities and observed outcomes, with lower values indicating better calibrated and more accurate predictions. The first is a degenerate model that always predicts a trustworthiness of 1.0. The second predicts a constant value equal to the mean of each chemist's empirical trust rate; this baseline is calibrated to the overall disagreement rate but non‐discriminative, and therefore any useful trust metric should improve upon it. As shown in the right panel of Figure 4, the AIF trustworthiness score achieves consistently lower Brier scores than both constant baselines. This demonstrates that the trustworthiness score is meaningfully calibrated: it assigns high confidence to interpretations chemists tend to trust and lower confidence in ambiguous or contested cases, reflecting genuine uncertainty rather than overconfidence.

### 3.2 Evaluation on Precursor Genome Dataset

We next evaluated AIF on the Precursor Genome dataset following the protocol described in Section 2.3.2. As previously described, expert reassessments were performed for 80 selected samples, evenly split between cases where the lowest‐$R_{\mathrm{wp}}$ interpretation was originally judged satisfactory and unsatisfactory. Sample details are provided in Section S12.2 (Tables S3, S4, and S5).

#### Agreement Cohort

**FIGURE 5:**
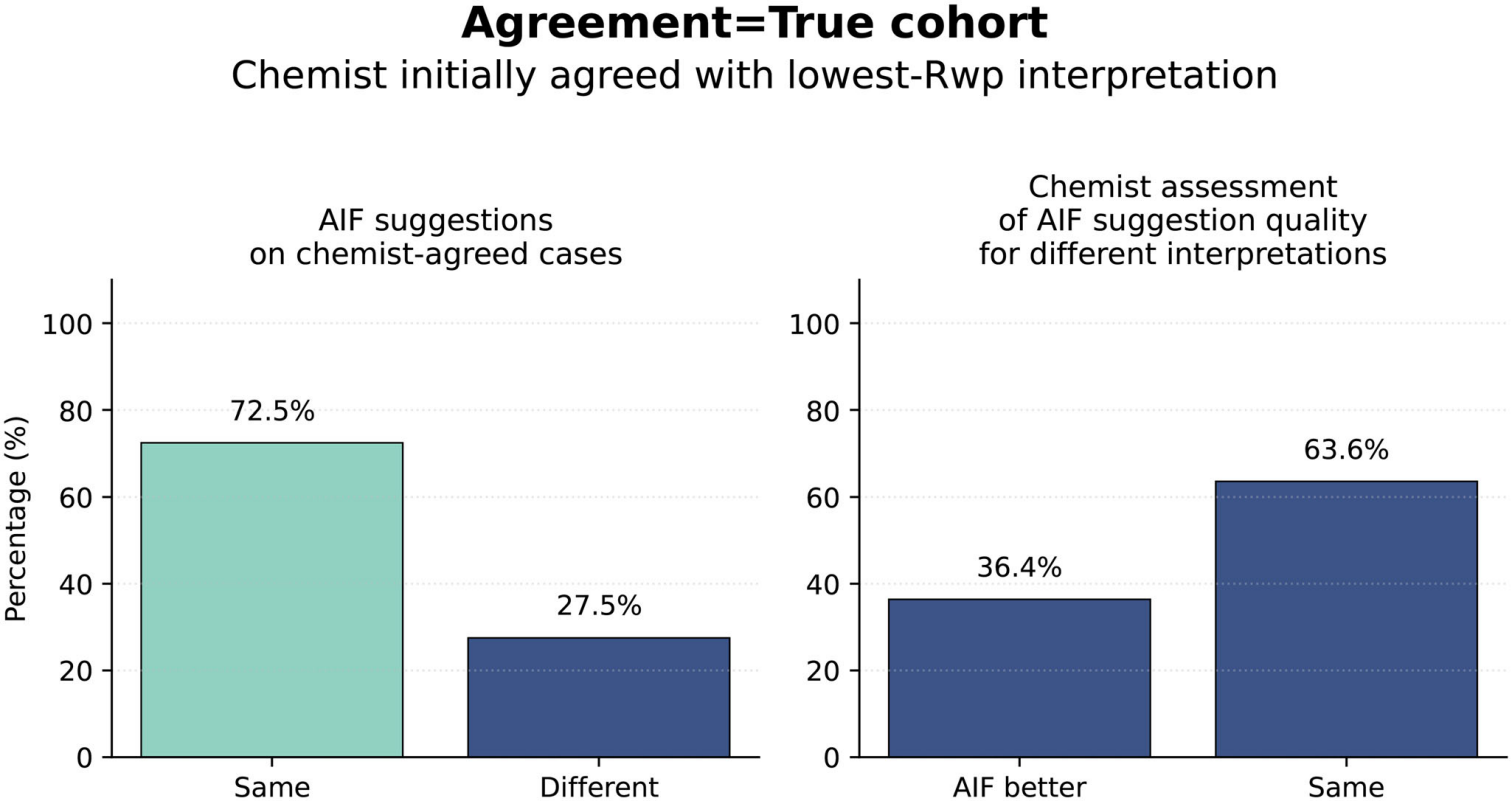
Summary of AIF behavior on the agreement cohort. Left: frequency with which AIF agreed with the lowest‐$R_{\mathrm{wp}}$ interpretation versus proposing an alternative. Right: expert assessment of AIF's alternative when it differed from the lowest‐$R_{\mathrm{wp}}$ result.

Figure 5 summarizes the results for samples in which the expert originally agreed with the lowest‐$R_{\mathrm{wp}}$ interpretation. In most of these cases, AIF reproduced the same interpretation (72.5%). When AIF proposed an alternative (27.5%), the expert judged the AIF interpretation to be better in 36.4% of cases, and equivalent in the remainder. Importantly, the expert did not once prefer the originally evaluated interpretation with the lowest‐Rwp when AIF suggested a different solution, indicating that AIF generally preserves correctness in this cohort.

#### Disagreement Cohort

**FIGURE 6:**
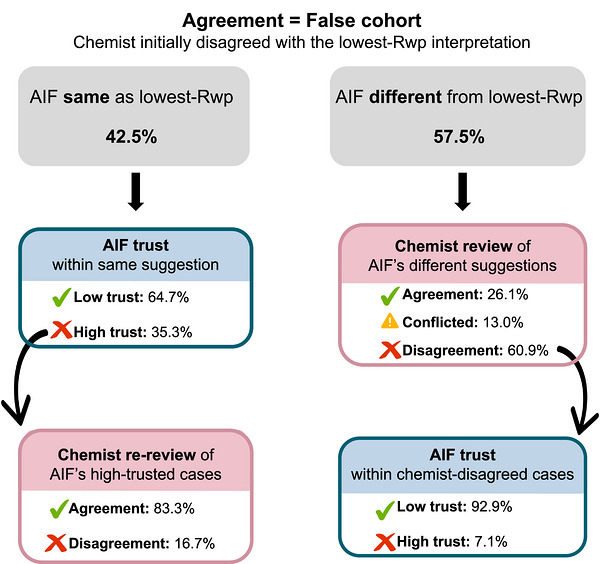
Summary of AIF behavior in the disagreement cohort of the Precursor Genome dataset (samples for which the expert initially judged the lowest‐$R_{\mathrm{wp}}$ refinement to be unsatisfactory). The diagram shows the fraction of cases in which AIF re‐selects the lowest‐$R_{\mathrm{wp}}$ interpretation versus proposes an alternative, followed by expert re‐evaluation and AIF trustworthiness scores within each branch.

Figure 6 shows the results for the samples where the expert originally deemed the lowest‐$R_{\mathrm{wp}}$ refinement unsatisfactory. In this disagreement cohort, AIF reproduced the original lowest‐$R_{\mathrm{wp}}$ interpretation in 42.5% of cases, while proposing an alternative interpretation in the remaining 57.5%. This elevated rate of alternative proposals, relative to the agreement cohort, reflects AIF's tendency to diverge from structure model interpretations previously judged problematic.

In the cases in which AIF reproduced the original lowest‐$R_{\mathrm{wp}}$ interpretation, the trustworthiness score was low in 64.7% of cases (consistent with the original chemist evaluation) and high for the remaining 35.3%. Because this subset was non‐negligible, we asked the expert annotator to re‐evaluate these high‐trust cases; during this reassessment, the annotator was aware that these samples corresponded to cases initially deemed unsatisfactory for which AIF assigned high trust to the lowest‐$R_{\mathrm{wp}}$ interpretation. Upon re‐review, the annotator revised their original assessment in 83.3% of them and ultimately agreed with the AIF/lowest‐$R_{\mathrm{wp}}$ interpretation. To assess whether this result was influenced by confirmation bias, an independent expert who was not involved in the original Precursor Genome evaluation performed a blinded re‐evaluation of 17 samples from the false cohort (6 high‐trust and 11 low‐trust) in randomized order, without knowledge of AIF's trust scores. Overall agreement between the independent evaluator and AIF's trust assignments was 76.5% (13/17), with symmetric disagreements: in two cases the independent evaluator trusted an interpretation that AIF flagged as low‐trust, and in two cases the reverse. These results support that the 83.3% reversal rate reflects genuine re‐assessment rather than systematic bias introduced by awareness of AIF's trust scores.

Among the cases in which AIF proposed an alternative interpretation, the expert agreed with the AIF interpretation in 26.1% of cases and disagreed in 60.9%. A smaller but notable fraction of 13.0% produced a “conflicted” outcome, where the expert reassessed their original judgment after seeing the AIF suggestion. Such cases reflect the inherent ambiguity of certain diffraction patterns and the practical variability introduced during manual refinement. Providing multiple, probabilistically ranked alternatives helps surface these ambiguities and reduce the risk that single‐solution workflows propagate unnoticed mistakes.

The trustworthiness score provides a complementary lens for interpreting these outcomes, incorporating data‐quality and refinement–stability diagnostics that do not enter the posterior alongside the chemistry priors that do. Among the cases in which the expert rejected AIF's suggested alternative (60.9% of samples), AIF assigned low trust to its own recommendation in 92.9% of cases, indicating that the framework generally recognizes when its suggestions are weakly supported. Importantly, these samples belong to the disagreement cohort precisely because the expert had already deemed the lowest‐$R_{\mathrm{wp}}$ interpretation unsatisfactory, meaning there is no acceptable baseline to compare against in these cases. The 60.9% rejection rate therefore does not reflect AIF proposing worse solutions than the baseline; rather, it reflects a subset of samples for which no interpretation in the candidate set adequately describes the diffraction pattern, a limitation of the upstream hypothesis generation step rather than of the scoring or trust logic.

Together, these results indicate that the trustworthiness score not only suppresses overconfident recommendations, but can also flag cases in which an initial human judgment may have been overly conservative or mistaken. A complementary quantitative evaluation of trust calibration, including Brier score analysis and comparisons against baseline confidence measures, is provided in Section S12.3.

### 3.3 Case Study: Chemically Implausible Interpretation Rejected Through Chemical Reasoning

The following case study illustrates how probabilistic ranking combined with chemically‐informed priors resolves ambiguities that cannot be addressed through diffraction‐fit metrics alone. The case studies were deliberately chosen so that the correct interpretation is fairly routine to a domain expert. This is appropriate to the framework's intended contribution: automating standard, textbook‐level chemical reasoning at the scale required for autonomous laboratories, rather than performing novel chemical inference. A second case study where the correct polymorph identified through chemical reasoning is presented in Section S13.2.

A representative case arises from a synthesis targeting ${\mathrm{CaVO}}_{2}$, using ${\mathrm{CaCO}}_{3}$ (space group 167) and $V_{2}$ $O_{5}$ (space group 59) as precursors at a 1:1 stoichiometric ratio of Ca:V, and heated at 600 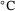 for 12 h in a tube furnace under an Ar+2%$H_{2}$ atmosphere. Among the four candidate interpretations generated for the resulting diffraction pattern, the interpretation with the lowest $R_{\mathrm{wp}}$ (Interpretation 1) includes ${\mathrm{CaC}}_{2}$, a phase that is chemically unrealistic under these conditions. Although ${\mathrm{CaC}}_{2}$ is a well‐known industrial compound, its formation requires extremely reducing, carbon‐rich environments. Under the present synthesis conditions, carbon is present only as carbonate, which is expected to decomposes to CaO with release of ${\mathrm{CO}}_{2}$. Moreover, the presence of oxygen‐bearing precursors and moderate synthesis temperature strongly disfavors carbide formation, rendering ${\mathrm{CaC}}_{2}$ chemically unlikely despite a favorable numerical fit by refinement in the recovered PXRD pattern.

Indeed, Interpretation 1 achieves both a relatively low $R_{\mathrm{wp}}$ value for the scan parameters (16.16) and a near‐perfect composition‐balance score based on weight fractions recovered from refinement of suggested phases (0.998 out of 1). This underscores how purely numerical figures of merit can favor chemically implausible solutions. The LLM‐derived chemical prior, however, penalizes ${\mathrm{CaC}}_{2}$ with a very low phase likelihood (0.1 out of 1) in every interpretation in which it appears. This phase‐level assessment differs from the LLM's interpretation‐level likelihood (e.g., 0.4 for Interpretation 1), which reflects the plausibility of the phase assemblage as a whole rather than any single constituent. For ${\mathrm{CaC}}_{2}$, the LLM explanation is:

“$CaC_{2}$ is highly unlikely to form under these conditions due to the presence of oxygen in the atmosphere, which would oxidize any carbon present. The formation of ${\mathrm{CaC}}_{2}$ would require a reducing environment, which is not provided here. The Ar+$H_{2}$ atmosphere does not favor carbide formation from ${\mathrm{CaCO}}_{3}$ and $V_{2}$ $O_{5}$ at 600 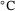
”

**FIGURE 7:**
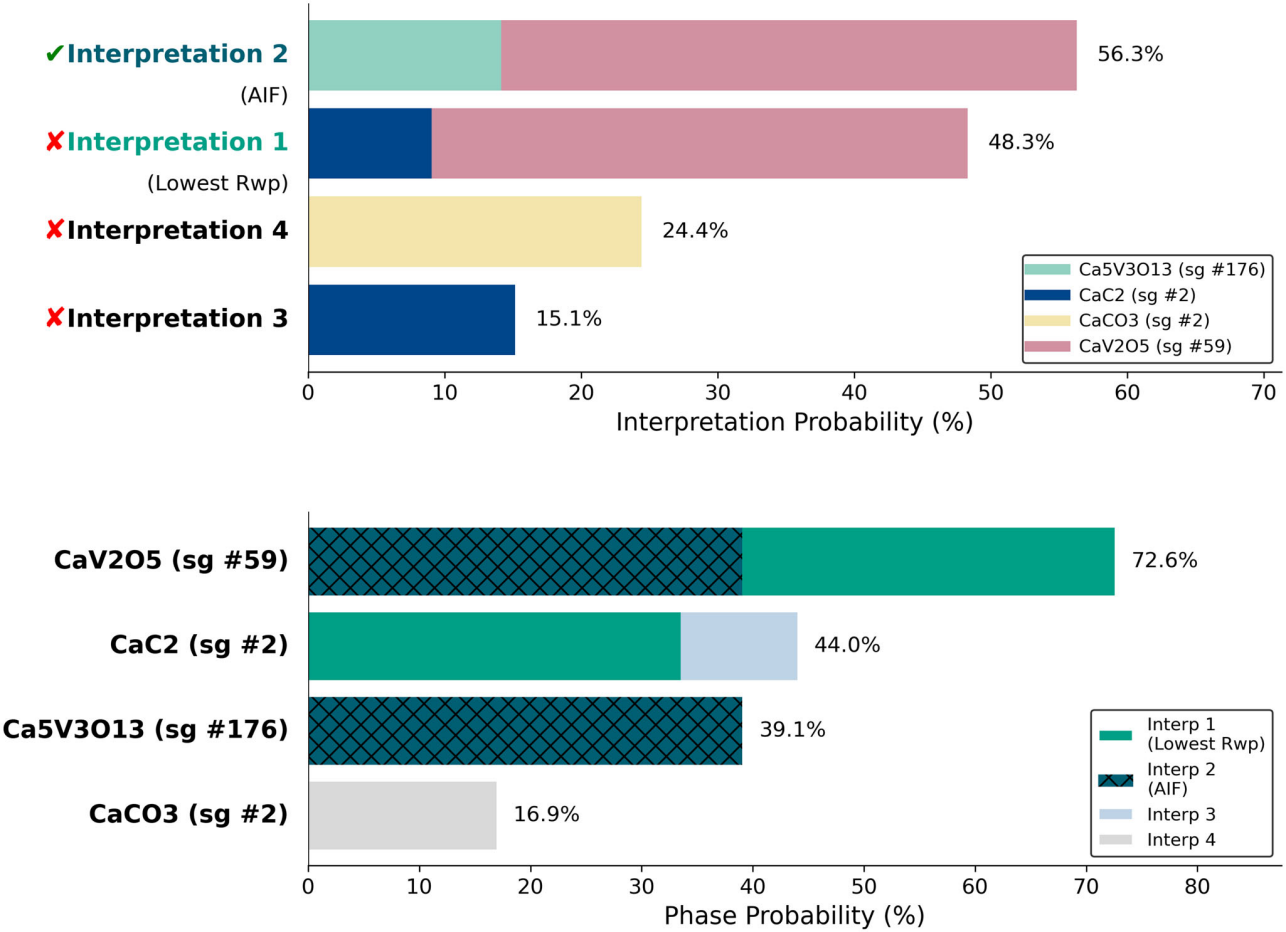
Probabilistic output of AIF for the ${\mathrm{CaVO}}_{2}$ synthesis case study. The top panel shows posterior probabilities of candidate interpretations, with colored segments indicating the refined weight fractions of the constituent phases. The bottom panel shows phase‐level posterior probabilities, with colors indicating the contribution of each interpretation.

**FIGURE 8:**
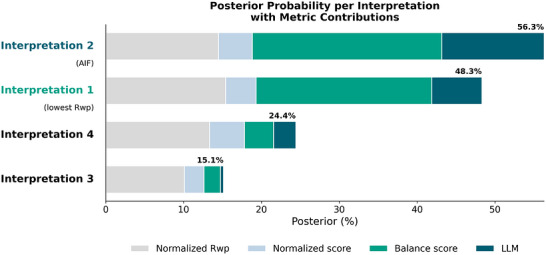
Metric‐level contribution breakdown for the ${\mathrm{CaVO}}_{2}$ synthesis case study. The chemically implausible Interpretation 1 receives large contributions from $R_{\mathrm{wp}}$ and balance score, but a strongly suppressive contribution from the LLM likelihood.

Figure 7 summarizes the resulting interpretation‐ and phase‐level posterior probabilities, while Figure 8 shows how each metric contributes to the final ranking, clarifying why the chemically implausible lowest‐$R_{\mathrm{wp}}$ solution is penalized.

To interpret these probabilistic outputs, recall that AIF assigns each candidate interpretation $I_{n}$ a posterior probability $P(I_{n}|D)$ according to Bayes' theorem (Equation 1). In the top panel of Figure 7, each horizontal bar corresponds to a candidate interpretation, with stacked segments proportional to the refined phase fractions of the constituent phases. The lowest‐$R_{\mathrm{wp}}$ interpretation is indicated by a teal label, whereas AIF's top‐ranked interpretation is shown in deep teal (hatched). Check marks indicate interpretations assessed as trustworthy by AIF's independent trustworthiness diagnostic, while crosses denote interpretations flagged as untrustworthy. In the bottom panel, phase‐level posterior probabilities $P(f_{i}|D)$ are obtained by marginalizing over interpretations (Equation 4); phases attain high probability when they appear consistently across high‐probability interpretations.

Interpretation 2, which contains ${\mathrm{CaV}}_{2}$ $O_{5}$ and ${\mathrm{Ca}}_{5}$ $V_{3}$ $O_{13}$ and excludes ${\mathrm{CaC}}_{2}$, receives a substantially higher LLM interpretation likelihood (0.75 out of 1) and is the only interpretation marked as trustworthy by AIF (highlighted with checkmark on the output interpretation probabilities). Although Interpretation 1 is competitive or superior on diffraction‐fit and composition‐balance metrics alone, the inclusion of ${\mathrm{CaC}}_{2}$ incurs a strong chemical plausibility penalty, which suppresses its overall posterior probability. As a result, AIF shifts probability toward Interpretation 2 and correspondingly reduces the inferred probability of ${\mathrm{CaC}}_{2}$ being present. A complete breakdown of the metric values for each candidate interpretation is provided in Table S6.

## 4 Discussion

This work demonstrates that automated phase identification from powder x‐ray diffraction benefits fundamentally from reframing interpretation as a probabilistic, chemistry‐aware inference problem rather than a deterministic optimization task. Across both blinded expert benchmarks and targeted case studies, AIF consistently produces interpretations that are more chemically plausible than those suggested by a single figure of merit to describe fit agreement alone, such as the lowest‐$R_{\mathrm{wp}}$. As shown in the multi‐project evaluation, even experienced chemists often disagree on the most credible interpretation, and a substantial fraction of experimental PXRD patterns admit no clearly acceptable solution. In this regime, small numerical differences in refinement metrics do not reliably correspond to physically meaningful distinctions, limiting the effectiveness of purely deterministic decision rules. By explicitly integrating diffraction quality with chemical reasoning based on LLMs, AIF yields rankings that better reflect researcher judgment while preserving uncertainty where the data do not support a confident conclusion.

The case studies highlight two archetypal failure modes of lowest‐$R_{\mathrm{wp}}$ selection and illustrate how chemically informed probabilistic reasoning resolves them. In the first, a numerically favorable refinement favors a chemically implausible phase, which is only rejected once chemical plausibility is explicitly considered. In the second, multiple interpretations fit the data nearly equally well, and discrimination requires knowledge of phase stability and polymorphism that is not encoded in refinement metrics. In both cases, AIF does not override diffraction fitting; rather, it constrains interpretation to chemically coherent explanations and downweights hypotheses that contradict well‐established chemical knowledge. Equally important, when no candidate interpretation is strongly supported, the framework preserves and exposes this ambiguity instead of forcing a spurious single answer.

The case studies presented here are deliberately drawn from chemistries familiar to a trained solid‐state chemist (carbide formation under oxidising conditions; high‐temperature $V_{2}$ $O_{3}$ polymorph stability). This is the appropriate evidentiary standard for what AIF is designed to do: automate the routine chemical reasoning that competent chemists already apply by hand, at the scale required by autonomous laboratories.

The LLM‐derived plausibility score is weighted below $R_{wp}$ but equal to the peak‐matching contribution, and is intended to be decisive in chemically ambiguous cases where the refinement‐based metrics do not discriminate among candidates. Importantly, these LLM likelihoods are not simply opaque numerical outputs: the model includes human‐readable rationales grounded in synthesis conditions, phase stability, redox chemistry, and polymorphism. This interpretability enables direct inspection of why specific phases are penalized or favored, as illustrated in the case studies, and distinguishes the approach from prior machine‐learning methods that infer plausibility solely from latent numerical features. More broadly, this work shows that recent advances in large language models make it possible to programmatically encode aspects of chemical intuition that were effectively inaccessible to automated systems only a few years ago.

At the same time, several limitations and open directions remain. We note that the 20‐sample multi‐project benchmark, evaluated by four chemists, is statistically modest: individual decision flips materially affect the reported point estimates, and the confidence intervals reported in Section 3.1, are correspondingly wide. Expanding the held‐out test set is a near‐term priority for future work.

The quality of LLM‐based reasoning depends on prompt design and underlying model capabilities [36], and outputs may vary across model versions or repeated evaluations. While we mitigate this by conservatively weighting the LLM prior and combining it with independent physical and chemical metrics, rare phases, unusual oxidation states, or non‐equilibrium products may still be undervalued. Moreover, the current implementation relies on ChatGPT, a closed‐source model. Initial tests with the open‐source Llama4 Scout model show qualitatively similar trends but lower overall reliability (approximately 60%), consistent with Scout being a comparatively small model optimized for efficiency rather than deep domain reasoning (Section S10.1). Broader evaluation of larger, frontier‐scale open models will therefore be essential to assess robustness, reproducibility, and chemical reasoning performance across model families. This is the case both for our specific application as well as broader usage of LLMs to direct tasks in chemistry and materials science [37, 38, 39, 40, 41].

A further consideration is the relationship between the LLM's training corpus and the data on which AIF was evaluated. We audited the three data sources against publicly released supplementary data for the A‐Lab Nature paper [1] and against the prior Montoya et al. study [42] that established the TRI methodology. Of the 20 samples in the multi‐project test set, 6 (carrying five distinct target compositions) appear in the A‐Lab paper's public synthesis‐results release (${\mathrm{CaTiNiP}}_{2}$ $O_{9}$, ${\mathrm{MgCuP}}_{2}$ $O_{7}$, ${\mathrm{MnAgO}}_{2}$, ${\mathrm{Ta}}_{4}$ ${\mathrm{PbO}}_{11}$, and $Y_{3}$ ${\mathrm{In}}_{2}$ ${\mathrm{Ga}}_{3}$ $O_{12}$), accompanied in that release by refinement‐result images with the identified phases labeled in each plot legend; the raw diffraction patterns and the alternative candidate interpretations evaluated by AIF here were not part of that release. The remaining 14 samples, 11 TRI new‐compounds samples covering Ca‐, Li‐, Na‐, K‐, and Mg‐vanadate chemistries, two Precursor Genome samples, and one A‐Lab sample (${\mathrm{BaMn}}_{8}$ $O_{16}$), do not appear in any public release to the best of our knowledge. Furthermore, to test whether potential data exposure affected performance, we scored each of the 20 test‐set samples by the number of expert judgments (out of four) rating the AIF‐derived result as best or tied (i.e., “AIF” or “both”), since data leakage, if present, would manifest as an inflated rate of such AIF‐favorable judgments. We compared the six potentially exposed samples against the remaining fourteen using an exact two‐sided Mann–Whitney U test on the per‐sample scores. The AIF result was rated best or tied in 13/24 judgments (54%) for the potentially exposed samples versus 35/56 (63%) for the unexposed samples, a difference that is not statistically significant (U=38, p=0.78). We note that the direction of this (non‐significant) difference is opposite to what data contamination would predict, as the potentially exposed samples performed slightly worse rather than better.

Several alternative chemistry‐aware methods address related problems in PXRD interpretation, including human‐in‐the‐loop Bayesian phase mapping, [23] probabilistic deep‐learning classification of multi‐phase patterns, [13] and Bayesian algorithm execution for targeted acquisition [24]. These methods operate on different inputs and address adjacent tasks, whereas AIF re‐ranks an existing pool of candidate interpretations; the approaches are therefore complementary, and AIF could be applied to re‐rank the candidate outputs of these frameworks. A corresponding limitation of our evaluation is that both compared interpretations draw from the same upstream candidate set: the evaluation establishes that AIF improves ranking over the lowest‐$R_{\mathrm{wp}}$ default, not that it recovers correct interpretations when the candidate pool itself is deficient, as reflected in the high rejection rate in the Precursor Genome disagreement cohort.

An additional practical consideration is transferability across experimental settings. The thresholds, weights, and diagnostic criteria used in AIF are calibrated for the diffraction data quality, noise characteristics, and synthesis workflows examined within the A‐Lab [1] laboratory. Different laboratory setups, instrument configurations, signal‐to‐noise levels, or user preferences may require recalibration of metric weights or trustworthiness thresholds to maintain appropriate sensitivity and specificity. While the probabilistic framework itself is general, deployment in new environments will likely involve lightweight recalibration of fitted parameters.

Finally, limitations arise when strong refinement metrics conflict with chemical knowledge not captured by the current descriptors, such as kinetic constraints, phase‐diagram boundaries, or microstructural considerations. Extending the framework to incorporate additional domain priors  [43]—such as thermodynamic stability from first‐principles calculations [44], constraints on refined strain or microstructure parameters, additional figures of merit such as the statistical fit parameter $\chi^{2}$, or explicit kinetic models [45]—represents a natural next step. More generally, future work might explore whether the same principles could be applied to other characterization techniques where expert interpretation and chemical intuition remain bottlenecks  [46].

## 5 Conclusion and Future Directions

We have introduced the automated interpretation framework (AIF), a probabilistic and chemically informed approach to phase identification that moves beyond deterministic selection based on purely fit‐based metrics such as lowest‐$R_{\mathrm{wp}}$. Instead, AIF incorporates chemical reasoning through both numerical scores (such as composition balance) as well as chemical intuition from state‐of‐the‐art LLMs. Across expert benchmarks and high‐throughput datasets, this approach corrects chemically implausible solutions and identifies cases where no interpretation is sufficiently supported for autonomous use. Looking forward, this framework provides a foundation for incorporating richer sources of chemical and physical knowledge, including thermodynamic and kinetic models, microstructure sensitive descriptors, and complementary characterization modalities (e.g., SEM–EDS). As autonomous and high‐throughput materials discovery continues to expand, such trust‐aware interpretation frameworks will be critical for closing the loop between synthesis, characterization, and decision‐making.

## Author Contributions

O.D. contributed to the conceptualization of the project, developed the software framework, performed the analyses, and wrote the original manuscript, including subsequent revisions and edits. A.J. contributed to project conceptualization, provided methodological guidance and supervision, contributed to writing and editing of the manuscript and conducted the expert review used to calibrate the relative weighting of AIF's evaluation metrics. L.W. contributed as an expert chemist in the multiproject evaluation and the Precursor Genome analysis and provided feedback and suggested edits during manuscript review. A.T., S.T., and A.V. contributed as expert chemists in the multiproject evaluation and provided feedback during framework development. L.J evaluated the model's results using Llama‐based model and G.C. reviewed the manuscript and provided scientific feedback.

## Conflicts of Interest

The authors declare no conflicts of interest.

## Supporting information


**Supporting File**: advs76450‐sup‐0001‐SuppMat.pdf.

## Data Availability

The full implementation of AIF is publicly available at https://github.com/hackingmaterials/AIF. The specific version used to generate all results reported in this manuscript corresponds to release v1.0 (https://github.com/hackingmaterials/AIF/releases/tag/v1.0). PXRD patterns used for evaluation were generated by A‐Lab [1], the autonomous laboratory for inorganic synthesis at LBNL. The framework used to select the lowest‐$R_{\mathrm{wp}}$ interpretations is Dara [26]. The chemical‐plausibility estimates were generated using ChatGPT 4o (OpenAI, 2024). For comparison, we also evaluated the open‐source Llama4 Scout model  [47]. The 80‐sample Precursor Genome re‐evaluation subset used in this study is enumerated by sample ID, target composition, and synthesis conditions in Tables S4 and S5, with details of the re‐evaluated cases in Table S3. The corresponding diffraction patterns and refinement outputs have been deposited as a versioned Zenodo archive with DOI: https://doi.org/10.5281/zenodo.21141588.
