# Supplementary material for: Automating Chemical Reasoning in High‐Throughput Phase Identification With a Probabilistic, LLM‐Guided Framework
Source: Adv Sci (Weinh). 2026 Aug 3:e76450. Online ahead of print. doi: 10.1002/advs.76450 (PMC13430547; doi:10.1002/advs.76450)
Supplement: Supplementary file 1 — Supporting File: advs76450‐sup‐0001‐SuppMat.pdf. [file ADVS-9999-e76450-s001.pdf]

Supplementary Information

9 Rietveld refinement parameters

Table S1 summarizes the parameters used in the Dara Rietveld refinement, including which quantities were refined and the constraints applied. Reference crystallographic information files (CIFs) used as starting structures for this analysis were obtained from the Inorganic Crystal Structure Database (ICSD).

**Parameter**	**Meaning**	**Refined?**	**Range / constraint**
a, b, c	Lattice lengths (nm)	Yes	±2 % (`lattice_range`=0.02)
α, β, γ	Lattice angles (°)	Yes	±2 %
k_1, k_2, b_1	Peak-shape parameters	Yes	k_1: 0–1; k_2: 0–0.001; b_1: 0–0.001
`EPS2`	Sample displacement	Yes	−0.05 to +0.05
`GrainSize`	Crystallite size (nm)	No	Fixed (initial value)
Atomic positions (x, y, z)	Atom coordinates	No	Fixed
`GEWICHT`	Phase scale factor	Yes	GEWICHT=SPHAR4 (Implicitly refined)

Table S1: Parameters used in the Rietveld refinement.

10 LLM prompt and hyper-parameters

Hyper-parameters: Model = `gpt-4o-2024-08-06`; Temperature = 0.
LLM is used to evaluate the likelihood of a phase being present based on synthesis conditions, as well as the overall likelihood of the interpretation. It provides a probability value between 0 and 1, reflecting the LLM's confidence in the phase(s)'s formation.

The LLM's output probability is generated using a few shot prompting technique, where five examples are provided to guide the model. In addition to numerical likelihoods, the LLM is instructed to return structured, human-readable explanations for each phase and interpretation, which are used to interpret model behavior and are referenced in the main text case studies. These examples illustrate the expected reasoning steps and output format, helping ensure consistent interpretation of synthesis conditions and stable probability estimates.

LLM scores were queried once per interpretation. During prompt development, scores were queried three times per interpretation and averaged; after prompt refinement, run-to-run variance was observed to be at most 0.1 (typically 0 or 0.05), and single-query scoring was adopted for efficiency. The prompt refinements that stabilized outputs were: (1) explicit instructions that interpretation likelihood reflects compatibility and completeness of the phase set as a whole rather than an average of individual phase likelihoods; (2) a consistency rule requiring phases with the same chemical identity to receive the same likelihood across interpretations; (3) a stability reminder instructing the model to use deterministic logic, synthesis rules, and known reaction windows to ground its scoring; and (4) five few-shot examples illustrating the expected reasoning steps and output format.

Prompt construction. The Python code used to construct the LLM input prompt is shown below:

```
prompt = textwrap.dedent(f"""
    Given the following synthesis data:
    {synthesis_data}

    Below are multiple proposed phase interpretations. For each
        interpretation, determine the likelihood that the listed solid phases
        have formed under the given synthesis conditions.

    Take into account:
    - Whether the oxidation state is thermodynamically plausible (based on
        precursors, temperature, and synthesis atmosphere).
    - Whether the specific polymorph (space group) is known to be stable at
        the synthesis temperature and pressure. If multiple polymorphs exist
        for the same composition, prefer the polymorph known to be stable
        under the synthesis conditions.
    - Whether the overall elemental composition of the phases, weighted by
        their fractions, matches the expected target composition.
        Interpretations with large elemental imbalances (e.g., excess or
        missing cations) should be penalized. Use the provided composition
        balance score as an indicator of this match.
""")

# Add interpretation info
prompt += "\nInterpretations:\n"
for name, phases in all_phases.items():
    prompt += f"- {name}: {', '.join(phases)}\n"

# Add composition-balance scores
prompt += "\nComposition balance scores:\n"
for name, score in composition_balance_scores.items():
    prompt += f"- {name}: {round(score, 3)}\n"

prompt += load_prompt_template("llm_prompt_template.txt")

response = openai.ChatCompletion.create(
    model=model,
    messages=[
        {"role": "system",
         "content": "You are an expert in material synthesis and phase
             prediction. "
                    "Use thermodynamics, kinetics, and polymorph knowledge to
                        evaluate stability and likelihood of observed phases."},
        {"role": "user", "content": prompt},
    ],
    temperature=0,
    seed=42,
)
content = response["choices"][0]["message"]["content"].strip()
```

Listing 1: Python code used to construct the LLM input prompt.

Prompt template. What follows is the structured prompt template provided to the LLM, including the instructions and few-shot examples used to compute phase-plausibility likelihoods.

Prompt template

Instructions:
Phase Likelihood Scoring:

- Score each **individual phase** from 0 to 1 (likelihood it has formed under the synthesis conditions).
- Penalize polymorphs that are not stable at the synthesis temperature (e.g., low-T polymorphs at high T), but do **not assign 0** unless they are physically implausible or thermodynamically forbidden.
- If a polymorph is less stable but still possible due to mechanisms such as kinetic trapping or low synthesis duration, assign reduced likelihood (e.g., 0.5).
- If a phase is a known precursor or may remain unreacted, assign moderate likelihood (e.g., 0.4–0.6). If it is particularly inert under these conditions, assign higher (0.7–0.8).
- If a phase is physically implausible (e.g., CO as a solid, a gas-phase species, or a high-energy transient), assign near-zero likelihood (0.0–0.1). This should always be penalized more than plausible phases.

Interpretation Likelihood (overall):

- This is **not an average** of individual phase likelihoods.
- Score the overall interpretation based on the **compatibility, plausibility, and completeness** of the phase set as a whole.
- If a phase is implausible, penalize the overall interpretation likelihood significantly.
- If likely phases are **missing**, reduce the interpretation likelihood even if the present phases are individually plausible. For example, if only one phase is present but synthesis conditions suggest a multiphase product, reduce the score accordingly.
- Respect the reported weight fractions when considering importance or impact on interpretation. Phases with higher weight fractions must contribute proportionally more to the interpretation likelihood — especially if their individual phase likelihood is low, they should heavily penalize the interpretation.
- Take into account on how likely is an interpretation the composition balance.

Phase and Interpretation Likelihoods – Critical Rule:

- When scoring or evaluating phase likelihoods, judge based on the approximately equal to identity, not the formula label.
- Phases with the same "approximately equal to" must be assigned the same phase likelihood, regardless of their formula or how they appear across interpretations.
- Do not penalize complex or unusual formulas if their "approximately equal to" matches a known plausible phase.
- When evaluating interpretation plausibility, consider the combined presence and total weight of all phases with the same approximately equal to identity.

Completeness Check – Requirement:

- Penalize interpretations that **omit plausible or expected phases** based on the synthesis conditions, but do **not assign 0** unless they are physically implausible or thermodynamically forbidden.

- Penalize single-phase interpretations if other by-products, unreacted precursors, or side products would also be expected under the conditions.
- If the list is incomplete (e.g., missing likely by-products or unreacted precursors), apply a modest penalty depending on severity.

Explanation Requirements:

- For **each phase**, provide:

  1. A list of reasons / factors for a reduced likelihood score, along with the strength of each factor.
  2. For example, if the **polymorph** is less favorable, explain **how much** this affects its likelihood (not just that it's less stable). Use labels where appropriate to classify polymorphs as:

     – **Ground-state**
     – **Metastable**
     – **Kinetically trapped**
     – **Unknown/ambiguous**

  3. Use temperature, redox potential, and kinetics to justify likelihood numerically where possible. For example, provide rationales for whether a particular oxidation state in the product might form given starting precursors and reaction conditions. Consider whether anion groups such as carbonates are likely to decompose or be retained.

Stability Reminder (important):

- Do NOT allow the same interpretation to vary significantly in likelihood across runs.
- Use deterministic logic, synthesis rules, and known reaction windows to ground your scoring.

When synthesis fields are nan (unspecified)

- Treat missing fields as unknown; do not invent temperatures, atmospheres, durations, or precursors.
- Base phase and interpretation judgments on general plausibility (common solid phases, mutual chemical compatibility, reported weight fractions, and labeled identities), not on specific condition thresholds.
- Avoid hard penalties that depend on the missing field(s). If a judgment would require that field (e.g., "high-T only"), express uncertainty instead of penalizing strongly.
- You may infer a broad regime from coexisting phases (e.g., reduced vs. oxidized), but state that this is an assumption and keep the reasoning qualitative.
- Always acknowledge the unknowns in the explanation (e.g., "Temperature and atmosphere unspecified; evaluated on general plausibility and phase compatibility.").
- Still respect reported weight fractions: large fractions of questionable phases should reduce the overall interpretation plausibility more than trace amounts.
- Never treat gas-phase species or non-crystalline entities as valid solid phases, even if other fields are unknown.

Finally, remember that these phase interpretations are from x-ray diffraction measurements and therefore correspond to solid-phase crystalline materials. Glassy materials or gas phase species will not be included in the interpretation.
Do not confuse gas-phase species (e.g., CO, O) with solid phases — disqualify them appropriately. Format your response as a dictionary where each interpretation key maps to a nested dictionary with:

- "Likelihoods": {{phase: value}}
- "Explanations": {{phase: explanation}}

- "Interpretation_Likelihood": float
- "Interpretation_Explanation": str

The following examples are provided for demonstration purposes:

- Example 1: Solid state synthesis: gram-quantity precursors are mixed and heated in a furnace. Target: ZrTiO4 Precursors: ZrO2, TiO2 Temperature: 1273.15 K (1000°C) Dwell Duration: 4.0 hours Furnace: Box furnace with ambient air

Interpretations:

- I_1: ZrTiO4 (space group 18, weight fraction 80.00%, fractional_composition = {'Zr': 0.1667, 'Ti': 0.1667, 'O': 0.6667}, approximately equal to ZrTiO4), ZrO2 (space group 14, weight fraction 5.00%, fractional_composition = {'Zr': 0.3333, 'O': 0.6667}, approximately equal to ZrO2), TiO2 (space group 141, weight fraction 6.00%, fractional_composition = {'Ti': 0.3333, 'O': 0.6667}, approximately equal to TiO2), Al2TiO5 (space group 63, weight fraction 9.00%, fractional_composition = {'Al': 0.25, 'Ti': 0.125, 'O': 0.625}, approximately equal to Al2TiO5)
- I_2: ZrTiO4 (space group 60, weight fraction 80.00%, fractional_composition = {'Zr': 0.1667, 'Ti': 0.1667, 'O': 0.6667}, approximately equal to ZrTiO4), ZrO2 (space group 14, weight fraction 20.00%, fractional_composition = {'Zr': 0.3333, 'O': 0.6667}, approximately equal to ZrO2)
- I_3: ZrTi2O6 (space group 14, weight fraction 85.00%, fractional_composition = {'Zr': 0.1111, 'Ti': 0.2222, 'O': 0.6667}, approximately equal to ZrTi2O6), TiO2 (space group 136, weight fraction 15.00%, fractional_composition = {'Ti': 0.3333, 'O': 0.6667}, approximately equal to TiO2)

Composition balance scores:

- I_1: 0.940
- I_2: 0.900
- I_3: 0.783

Expected likelihoods and explanations: { "I_1": { "Likelihoods": { "ZrTiO4 (space group 18)": 0.4, "ZrO2 (space group 14)": 0.85, "TiO2 (space group 141)": 0.2, "Al2TiO5 (space group 63)": 0.6 }, "Explanations": { "ZrTiO4 (space group 18)": "Space group 18 (P2) is not the correct structure for ZrTiO under high-temperature solid-state synthesis. The accepted structure is orthorhombic Pbcn (SG 60). Use of SG 18 suggests a misidentified or distorted variant.", "ZrO2 (space group 14)": "Monoclinic ZrO (P2/c, SG 14) is the correct and stable phase at 1000°C. Likely present as unreacted precursor or due to Zr-rich stoichiometry.", "TiO2 (space group 141)": "Anatase (I4/amd, SG 141) transforms to rutile ¿600–800°C. At 1000°C, rutile (SG 136) should dominate. Anatase is not expected unless kinetically trapped.", "Al2TiO5 (space group 63)": "Tialite (Cmcm, SG 63) forms from TiO and AlO at high temperatures. Likely present due to Al contamination from crucibles or milling." }, "Interpretation_Likelihood": 0.50, "Interpretation_Explanation": "Incorrect space group for ZrTiO and implausible persistence of anatase TiO reduce the credibility of this interpretation, though ZrO and AlTiO are chemically consistent with moderate weight fractions." },
"I_2": { "Likelihoods": { "ZrTiO4 (space group 60)": 0.95, "ZrO2 (space group 14)": 0.85 }, "Explanations": { "ZrTiO4 (space group 60)": "Pbcn (SG 60) is the correct and well-established structure for disordered high-T ZrTiO. Its formation is expected at 1000°C.", "ZrO2 (space group 14)": "Residual monoclinic ZrO is plausible as a remnant precursor, especially with a slight excess or incomplete reaction. 20% weight is reasonable." }, "Interpretation_Likelihood": 0.93, "Interpretation_Explanation": "This is the most plausible interpretation: ZrTiO is in its correct ground-state structure, and unreacted ZrO is expected. The interpretation aligns well with the synthesis conditions and stoichiometry." },
"I_3": { "Likelihoods": { "ZrTi2O6 (space group 14)": 0.3, "TiO2 (space group 136)": 0.9 }, "Explanations": { "ZrTi2O6 (space group 14)": "ZrTiO is a Ti-rich phase, unlikely to form under 1:1 Zr:Ti stoichiometry. SG 14 is also uncommon for this compound; Pbcn or other orthorhombic settings are more appropriate.", "TiO2 (space group 136)": "Rutile (SG 136) is the correct high-temperature TiO

polymorph and expected to persist if Ti is in excess or unreacted." }, "Interpretation_Likelihood": 0.45, "Interpretation_Explanation": "The inclusion of a Ti-rich phase (ZrTiO) makes this interpretation unlikely from the given precursors. Although rutile is plausible, the overall composition deviates from expected reaction products." } }

- Example 2: Solid state synthesis: gram-quantity precursors are mixed and heated in a furnace. Target: NaMnO2 Precursors: Na2CO3, MnO2 Temperature: 1143.15 K (870°C) Dwell Duration: 10.0 hours Furnace: Box furnace with ambient air

Interpretations:

- I_1: NaMnO2 (space group 12, weight fraction 80.00%, fractional_composition = {'Na': 0.25, 'Mn': 0.25, 'O': 0.5}, approximately equal to NaMnO2), Mn2O3 (space group 206, weight fraction 20.00%, fractional_composition = {'Mn': 0.4, 'O': 0.6}, approximately equal to Mn2O3)
- I_2: NaMnO2 (space group 14, weight fraction 90.00%, fractional_composition = {'Na': 0.25, 'Mn': 0.25, 'O': 0.5}, approximately equal to NaMnO2), Mn2O3 (space group 206, weight fraction 10.00%, fractional_composition = {'Mn': 0.4, 'O': 0.6}, approximately equal to Mn2O3)
- I_3: NaMnO2 (space group 166, weight fraction 70.00%, fractional_composition = {'Na': 0.25, 'Mn': 0.25, 'O': 0.5}, approximately equal to NaMnO2), Na4Mn9O18 (space group 12, weight fraction 30.00%, fractional_composition = {'Na': 0.129, 'Mn': 0.2903, 'O': 0.5806}, approximately equal to Na2Mn5O9)

Composition balance scores:

- I_1: 0.900
- I_2: 0.950
- I_3: 0.942

Expected likelihoods and explanations: { "I_1": { "Likelihoods": { "NaMnO2 (space group 12)": 0.95, "Mn2O3 (space group 206)": 0.75 }, "Explanations": { "NaMnO2 (space group 12)": "C2/m (SG 12) is the accepted high-temperature polymorph of NaMnO (O'3-type). It is thermodynamically stable at ~870°C in air and expected to be the dominant phase from solid-state synthesis using NaCO and MnO.", "Mn2O3 (space group 206)": "MnO (Ia-3, SG 206) is a plausible secondary phase, forming from excess or unreacted MnO under oxidizing conditions. Its presence at 20% is reasonable in slightly Na-deficient or incomplete reactions." }, "Interpretation_Likelihood": 0.94, "Interpretation_Explanation": "This interpretation uses the correct NaMnO structure and includes a reasonable by-product. The phase set is chemically consistent, matches synthesis conditions, and aligns with the high composition balance score." },
"I_2": { "Likelihoods": { "NaMnO2 (space group 14)": 0.4, "Mn2O3 (space group 206)": 0.75 }, "Explanations": { "NaMnO2 (space group 14)": "P2/c (SG 14) is not the typical polymorph for NaMnO formed via solid-state reaction. This structure is associated with metastable or tunnel-type forms seen in low-T/hydrothermal methods. Its presence at 90% is unlikely under the given conditions.", "Mn2O3 (space group 206)": "As above, MnO is reasonable as a side product. Its 10% weight is modest and plausible." }, "Interpretation_Likelihood": 0.50, "Interpretation_Explanation": "Despite a strong balance score, the dominant NaMnO polymorph is inconsistent with the synthesis temperature and conditions, reducing overall credibility." },
"I_3": { "Likelihoods": { "NaMnO2 (space group 166)": 0.6, "Na4Mn9O18 (space group 12)": 0.2 }, "Explanations": { "NaMnO2 (space group 166)": "R-3m (SG 166) corresponds to the O3-type layered polymorph. It can occur under soft chemistry or electrochemical methods but is less stable than C2/m under high-T solid-state conditions. 70% is on the high side, but not impossible.", "Na4Mn9O18 (space group 12)": "This tunnel phase forms under ion-exchange or hydrothermal synthesis. Its formation at 870°C in solid-state air synthesis is highly unlikely, especially at 30% weight fraction." }, "Interpretation_Likelihood": 0.40, "Interpretation_Explanation": "The inclusion of NaMnO significantly lowers the plausibility of this interpretation. Although NaMnO R-3m is known, it is not the most stable under these synthesis conditions. Good balance score, but the phase set is not realistic." } }

- Example 3: Solid state synthesis: gram-quantity precursors are mixed and heated in a furnace. Target: MgFe2O4 Precursors: Fe2O3, MgO Temperature: 973.15 K (700°C) Dwell Duration: 12.0 hours Furnace: Box furnace with ambient air

Interpretations:

- I_1: MgFe2O4 (space group 62, weight fraction 80.00%, fractional_composition = {'Mg': 0.1429, 'Fe': 0.2857, 'O': 0.5714}, approximately equal to MgFe2O4), Fe2O3 (space group 74, weight fraction 15.00%, fractional_composition = {'Fe': 0.4, 'O': 0.6}, approximately equal to Fe2O3), Fe (space group 225, weight fraction 5.00%, fractional_composition = {'Fe': 1.0}, approximately equal to Fe)
- I_2: MgFe2O4 (space group 227, weight fraction 85.00%, fractional_composition = {'Mg': 0.1429, 'Fe': 0.2857, 'O': 0.5714}, approximately equal to MgFe2O4), Fe2O3 (space group 167, weight fraction 5.00%, fractional_composition = {'Fe': 0.4, 'O': 0.6}, approximately equal to Fe2O3), MgO (space group 225, weight fraction 5.00%, fractional_composition = {'Mg': 0.5, 'O': 0.5}, approximately equal to MgO), Fe (space group 229, weight fraction 5.00%, fractional_composition = {'Fe': 1.0}, approximately equal to Fe)
- I_3: MgFe2O4 (space group 227, weight fraction 98.00%, fractional_composition = {'Mg': 0.1429, 'Fe': 0.2857, 'O': 0.5714}, approximately equal to MgFe2O4), Fe (space group 225, weight fraction 2.00%, fractional_composition = {'Fe': 1.0}, approximately equal to Fe)

Composition balance scores:

- I_1: 0.934
- I_2: 1.000
- I_3: 0.994

Expected likelihoods and explanations: { "I_1": { "Likelihoods": { "MgFe2O4 (space group 62)": 0.5, "Fe2O3 (space group 74)": 0.3, "Fe (space group 225)": 0.05 }, "Explanations": { "MgFe2O4 (space group 62)": "Pnma (SG 62) is not the commonly stable spinel structure for MgFeO. Fd-3m (SG 227) is expected under these synthesis conditions. This may indicate a misassignment or distortion.", "Fe2O3 (space group 74)": "SG 74 (Pbnm) is not a known stable form for FeO. The expected polymorph is hematite (SG 167). Thus, this assignment is chemically questionable.", "Fe (space group 225)": "Metallic Fe (SG 225) is not stable in oxidizing environments like ambient air at 700°C. It should oxidize to FeO or remain absent." }, "Interpretation_Likelihood": 0.30, "Interpretation_Explanation": "Despite a good composition balance, this interpretation includes questionable polymorphs and an implausible metallic Fe phase, reducing overall confidence." },
"I_2": { "Likelihoods": { "MgFe2O4 (space group 227)": 0.95, "Fe2O3 (space group 167)": 0.9, "MgO (space group 225)": 0.8, "Fe (space group 229)": 0.05 }, "Explanations": { "MgFe2O4 (space group 227)": "Fd-3m (SG 227) is the correct and thermodynamically stable structure for MgFeO spinel. Formation is expected at 700°C in air.", "Fe2O3 (space group 167)": "Hematite (SG 167) is the correct and stable FeO polymorph under these conditions. A small residual amount is realistic.", "MgO (space group 225)": "Stable, inert oxide that may persist as a minor unreacted phase in Mg-rich or incompletely reacted systems.", "Fe (space group 229)": "Im-3m metallic Fe is unlikely to form or persist under oxidizing conditions; its inclusion is inconsistent with the synthesis atmosphere." }, "Interpretation_Likelihood": 0.70, "Interpretation_Explanation": "This interpretation includes three plausible phases with realistic weight fractions. Metallic Fe remains the only implausible component, slightly lowering the overall score despite a perfect composition balance." },
"I_3": { "Likelihoods": { "MgFe2O4 (space group 227)": 0.95, "Fe (space group 225)": 0.05 }, "Explanations": { "MgFe2O4 (space group 227)": "This is the expected spinel structure for MgFeO at 700°C in air, and it dominates the phase composition. The weight fraction is consistent with nearly complete reaction.", "Fe (space group 225)": "As in other cases, metallic Fe is not expected to survive under oxidizing conditions. Its presence is questionable but minimally weighted." }, "Interpretation_Likelihood": 0.80, "Interpretation_Explanation": "This interpretation has high chemical plausibility due to correct phase identification and a very low amount of metallic Fe, which is the only questionable component. Excellent composition balance reinforces its credibility." } }

- Example 4: Solid state synthesis: gram-quantity precursors are mixed and heated in a furnace. Target: Ti3O5 Precursors: TiO2, C7H6O2 Temperature: 1523.15 K (1250°C) Dwell Duration: 4.0 hours Furnace: Tube furnace with flowing Argon (flow rate unknown)

Interpretations:

- I_1: Ti3O5 (space group 12, weight fraction 98.00%, fractional_composition = {'Ti': 0.375, 'O': 0.625}, approximately equal to Ti3O5), Ti2O3 (space group 167, weight fraction 2.00%, fractional_composition = {'Ti': 0.4, 'O': 0.6}, approximately equal to Ti2O3)
- I_2: TiO (space group 225, weight fraction 20.00%, fractional_composition = {'Ti': 0.5, 'O': 0.5}, approximately equal to TiO), TiC (space group 225, weight fraction 35.00%, fractional_composition = {'Ti': 0.5, 'C': 0.5}, approximately equal to TiC), Ti3O5 (space group 12, weight fraction 45.00%, fractional_composition = {'Ti': 0.375, 'O': 0.625}, approximately equal to Ti3O5)
- I_3: Ti3O5 (space group 15, weight fraction 70.00%, fractional_composition = {'Ti': 0.375, 'O': 0.625}, approximately equal to Ti3O5), Ti4O7 (space group 2, weight fraction 30.00%, fractional_composition = {'Ti': 0.3636, 'O': 0.6364}, approximately equal to Ti4O7)

Composition balance scores:

- I_1: 1.000
- I_2: 1.000
- I_3: 1.000

Expected likelihoods and explanations: { "I_1": { "Likelihoods": { "Ti3O5 (space group 12)": 0.95, "Ti2O3 (space group 167)": 0.6 }, "Explanations": { "Ti3O5 (space group 12)": "P2/c (SG 12) is a well-characterized high-temperature polymorph of TiO, especially under reducing atmospheres. Its formation at 1250°C is highly plausible.", "Ti2O3 (space group 167)": "R-3c TiO is a known reduced titanium oxide and may form in small amounts if reduction proceeds further than TiO. Its 2% weight fraction is minor but chemically plausible under C-rich conditions." }, "Interpretation_Likelihood": 0.94, "Interpretation_Explanation": "A chemically coherent and structurally reasonable interpretation: the dominant TiO polymorph and a small amount of TiO are consistent with reducing synthesis in Ar and near-stoichiometric control." },
"I_2": { "Likelihoods": { "TiO (space group 225)": 0.5, "TiC (space group 225)": 0.35, "Ti3O5 (space group 12)": 0.95 }, "Explanations": { "TiO (space group 225)": "Rock salt TiO forms under very strong reducing conditions, requiring high oxygen deficiency. At 1250°C with CHO, it's possible but 20% is moderately high unless carbon excess is substantial.", "TiC (space group 225)": "TiC may form from carbothermal reduction if significant free carbon remains. 35% is only plausible if there is excess reductant and the system is oxygen-deficient.", "Ti3O5 (space group 12)": "Same as I_1 — P2/c TiO is expected under these synthesis conditions." }, "Interpretation_Likelihood": 0.78, "Interpretation_Explanation": "This interpretation includes reasonable phases, but the high combined weight of TiC and TiO (55%) requires unusually reducing conditions. If that level of reduction occurred, the presence of TiO would likely be suppressed." },
"I_3": { "Likelihoods": { "Ti3O5 (space group 15)": 0.6, "Ti4O7 (space group 2)": 0.85 }, "Explanations": { "Ti3O5 (space group 15)": "C2/c (SG 15) is less commonly reported for TiO and may reflect a metastable or less favorable polymorph. 70% is optimistic unless there is specific evidence for this variant.", "Ti4O7 (space group 2)": "TiO is a Magnéli phase that forms under reducing conditions and high temperatures. 30% is plausible, especially if reduction proceeded slightly beyond TiO." }, "Interpretation_Likelihood": 0.76, "Interpretation_Explanation": "This interpretation includes well-known reduced phases consistent with the synthesis conditions, but the dominant TiO polymorph is questionable. TiO formation is plausible, especially in carbon-rich environments." } }

- Example 5: Solid state synthesis: gram-quantity precursors are mixed and heated in a furnace. Target: NiFe2O4 Precursors: NiO, Fe2O3 Temperature: 1623.15 K (1350°C) Dwell Duration: 6.0 hours Furnace: Box furnace with ambient air

Interpretations:

- I_1: NiFe2O4 (space group 227, weight fraction 20.00%, fractional_composition = {'Ni': 0.1429, 'Fe': 0.2857, 'O': 0.5714}, approximately equal to NiFe2O4), MgFeAlO4 (space group 227, weight fraction 2.00%, fractional_composition = {'Mg': 0.1429, 'Fe': 0.1429, 'Al': 0.1429, 'O': 0.5714}, approximately equal to MgFeAlO4), MgFe2O4 (space group 227, weight fraction 30.00%, fractional_composition = {'Mg': 0.1429, 'Fe': 0.2857, 'O': 0.5714}, approximately equal to MgFe2O4), Fe2SiO4 (space group 62, weight fraction 48.00%, fractional_composition = {'Fe': 0.2857, 'Si': 0.1429, 'O': 0.5714}, approximately equal to Fe2SiO4)
- I_2: NiFe2O4 (space group 227, weight fraction 90.00%, fractional_composition = {'Ni': 0.1429, 'Fe': 0.2857, 'O': 0.5714}, approximately equal to NiFe2O4), NiO (space group 225, weight fraction 10.00%, fractional_composition = {'Ni': 0.5, 'O': 0.5}, approximately equal to NiO)
- I_3: NiFe2O4 (space group 227, weight fraction 70.00%, fractional_composition = {'Ni': 0.1429, 'Fe': 0.2857, 'O': 0.5714}, approximately equal to NiFe2O4), Fe2O3 (space group 167, weight fraction 20.00%, fractional_composition = {'Fe': 0.4, 'O': 0.6}, approximately equal to Fe2O3), NiO (space group 225, weight fraction 10.00%, fractional_composition = {'Ni': 0.5, 'O': 0.5}, approximately equal to NiO)

Composition balance scores:

- I_1: 0.727
- I_2: 0.933
- I_3: 1.000

Expected likelihoods and explanations: { "I_1": { "Likelihoods": { "NiFe2O4 (space group 227)": 0.9, "MgFeAlO4 (space group 227)": 0.05, "MgFe2O4 (space group 227)": 0.1, "Fe2SiO4 (space group 62)": 0.05 }, "Explanations": { "NiFe2O4 (space group 227)": "The normal spinel phase (Fd-3m, SG 227) is the ground-state structure of NiFeO and is expected to form under the synthesis conditions. However, the very low weight fraction (20%) is not consistent with targeting this as the main product.", "MgFeAlO4 (space group 227)": "Unlikely unless contamination from alumina crucibles or grinding media occurred. 2% is trace-level and marginally plausible, but should be flagged.", "MgFe2O4 (space group 227)": "Magnesium ferrite is plausible only with Mg contamination. A 30% presence implies major contamination, which is not expected given the listed precursors.", "Fe2SiO4 (space group 62)": "This olivine phase implies significant Si contamination, likely from the crucible or environment. A 48% weight fraction is chemically inconsistent with the expected products." }, "Interpretation_Likelihood": 0.05, "Interpretation_Explanation": "Although NiFeO is present, its low weight fraction and the dominance of unlikely or contaminant phases make this interpretation implausible. Most of the composition is inconsistent with the synthesis target." },
"I_2": { "Likelihoods": { "NiFe2O4 (space group 227)": 0.95, "NiO (space group 225)": 0.6 }, "Explanations": { "NiFe2O4 (space group 227)": "This is the correct and stable spinel structure for NiFeO and is expected to form under high-temperature conditions in air. A 90% yield is consistent with near-complete reaction.", "NiO (space group 225)": "NiO is a precursor and may persist at grain boundaries or due to incomplete reaction. Its 10% weight is reasonable, especially given the moderate dwell time." }, "Interpretation_Likelihood": 0.85, "Interpretation_Explanation": "A highly plausible interpretation, though the absence of FeO suggests incomplete capture of possible unreacted precursors. Still, NiFeO dominates, and NiO residue is acceptable." },
"I_3": { "Likelihoods": { "NiFe2O4 (space group 227)": 0.9, "Fe2O3 (space group 167)": 0.6, "NiO (space group 225)": 0.6 }, "Explanations": { "NiFe2O4 (space group 227)": "Same as I_2: this is the expected main product under the synthesis conditions, and a 70% yield is realistic.", "Fe2O3 (space group 167)": "FeO (R-3c, SG 167) is a stable precursor and may remain unreacted. 20% weight fraction is plausible, especially with diffusion-limited kinetics.", "NiO (space group 225)": "As a solid precursor, NiO may persist in small amounts. Its presence alongside FeO indicates partial reaction and is consistent with solid-state synthesis behavior." }, "Interpretation_Likelihood": 0.95, "Interpretation_Explanation": "This is the most chemically and compositionally plausible interpretation. NiFeO is present in high yield, and both NiO and FeO are reasonable unreacted residues. Combined with the perfect balance score, this is the most likely scenario." } }

10.1 LLM model comparison and prompt sensitivity

Because the interpretation-level likelihoods can influence the ranking in chemically ambiguous cases, we examined how consistently two different LLMs, ChatGPT and Llama, evaluate the same interpretations. This analysis also helps characterize the impact of model drift, since LLMs evolve over time and may not produce identical outputs across versions.

To quantify agreement, we measured the fraction of samples for which both models selected the same top-ranked interpretation within the AIF. Using the original prompt, ChatGPT and Llama agreed on 9 of the 15 samples (60%). After refining the prompt to reduce hallucinated structural modifications and enforce stricter output formatting, agreement increased to 10 of 15 samples (66.7%).

This improvement indicates that part of the model discrepancy originates from prompt sensitivity rather than fundamental chemical disagreement. Nonetheless, Llama's overall reliability remained appreciably lower than that of ChatGPT, consistent with the results reported in the main text. In particular, Llama was more prone to small hallucinated changes to interpretation metadata and to inconsistent scoring of metastable polymorphs. Prompt refinement mitigated but did not eliminate these issues.

Figure S1 summarizes the agreement before and after prompt refinement. Although the difference is modest, these results underscore the importance of prompt design when using LLM-based evaluation within autonomous PXRD workflows.

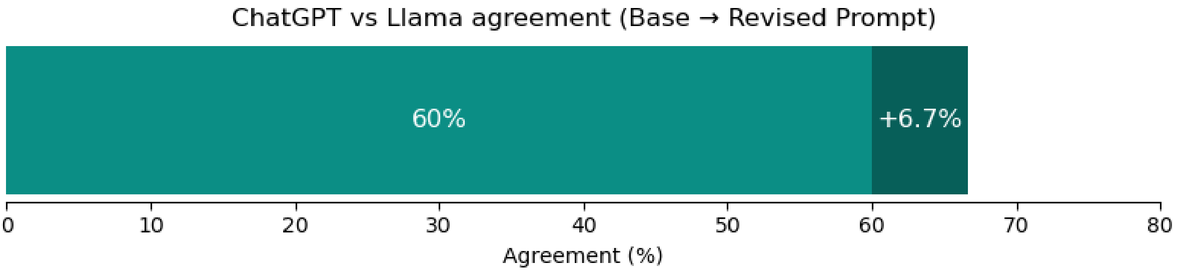

Figure S1: Agreement between ChatGPT and Llama. Using the base prompt, the two models selected the same top-ranked interpretation for 60% (9/15) of samples. After prompt refinement, agreement increased by 6.7 percentage points (to 66.7%), indicating that Llama's evaluations became more aligned with ChatGPT and fewer hallucinated changes to interpretation metadata occurred.

The prompt given to Llama for the LLM model comparison was iteratively revised to optimize agreement and minimize hallucinated structural modifications. This process was done by comparing responses using the original prompt to identify patterns in disagreement cases or hallucinations. If a pattern was found, a relevant section of the prompt template was identified and bullet points were added or revised to address this inconsistency. Throughout this process, an effort was made to maintain the original prompt structure.

The main revisions included a formatting reminder at the beginning of the prompt, scoring instruction bullet points in the "Phase and Interpretation Likelihoods" section, and editing the "Formatting Instructions" section for clarity.

Revised Prompt Template. Sections marked in bold indicate added or revised sections.

Revised Prompt Template

IMPORTANT
You are mixing phases from interpretation 2 into interpretation 1 in some samples. Include both. Then check Format Instructions for how to format your response.
Given the following synthesis data:

- ...

Instructions:

- ...

Phase Likelihood Scoring:

- ...

Interpretation Likelihood (overall):

- ...

Phase and Interpretation Likelihoods – Critical Rule:

- When scoring or evaluating phase likelihoods, judge based on the *approximately equal to* identity, not the formula label.
- Phases with the same *approximately equal to* must be assigned the same phase likelihood, regardless of their formula or how they appear across interpretations.
- Do not penalize complex or unusual formulas if their *approximately equal to* matches a known plausible phase.
- When evaluating interpretation plausibility, consider the combined presence and total weight of all phases with the same *approximately equal to* identity.
- **Do not copy, add, or infer phases from one interpretation into another.**
- **Phases with the same formula but different space groups must be treated as separate, unique phases.**
- **Penalize interpretations with gas-phase species (e.g. CO, O).**
- **Penalize unfavorable space groups and atmospheric incompatibility.**

Completeness Check – Requirement:

- ...

Explanation Requirements:

- ...

Stability Reminder (important):

- ...

When synthesis fields are nan (unspecified):

- ...

Format Instructions
Format your response as a dictionary where each interpretation key maps to a nested dictionary.
For ALL interpretations, provide:

- **I_#**: {
  - "Likelihoods": ...
  - ... }

The following examples are provided for demonstration purposes:

11 Metric engineering

11.1 Balance Score Calculation

The balance score provides a check on whether an interpretation preserves the overall elemental proportions implied by the synthesis recipe. Although a refinement may fit the diffraction pattern well, the proposed phases can still be chemically inconsistent—for example, by introducing elements not present in the precursors or by omitting elements that should be conserved.

To quantify this, the normalized elemental fractions of the target composition are compared with those obtained from the weighted set of output phases. Interpretations that closely conserve the expected elemental proportions receive scores near 1, while those that add or remove significant amounts of any element are penalized. This metric helps identify phases or phase combinations that are inconsistent with precursor chemistry or stoichiometric constraints.

In practice, the balance score is evaluated over a chosen set of elements that represent the conserved stoichiometric degrees of freedom for the target reaction. By default, we evaluate conservation over the elements present in the target composition, excluding elements whose inventory may change via exchange with the environment or volatilization (e.g. O, H, N, C), such as through gas-phase species like O_2, H_2O, CO, or CO_2. Missing target elements and extra elements not present in the target can be additionally penalized to discourage interpretations that violate conservation constraints.

$\text{Balance Score} = 1 - \sum_i \left| \text{Target}_i - \sum_j \left(\text{Weight Fraction}_j \times \text{Phase}_j(i) \right) \right|$

$\text{Balance Score} = 1 - \sum_i |\text{EF-Target}_i - \text{EF-Output}_i|$

- EF-Target_i = Normalized Elemental Fraction of element i in the **target composition**.
- EF-Output_i = Normalized Elemental Fraction of element i in the **combined output composition**.

11.2 Peak-match score

To rapidly assess the agreement between observed and simulated diffraction patterns prior to full refinement, we employ a heuristic peak-match score that compares peak positions and intensities. The score rewards agreement between observed and simulated peaks while penalizing missing or spurious simulated peaks, and is defined as

$$\text{Score} = \sum_i I_{\text{obs, matched}} + \sum_i I_{\text{obs, wrong intensity}} - 0.1 \sum_i I_{\text{sim, missing}} - 0.5 \sum_i I_{\text{sim, extra}}.$$

Here, $I_{\text{obs, matched}}$ denotes the intensities of observed peaks that are well matched in position and relative intensity by the simulated pattern. Peaks that are positionally matched but exhibit notable intensity discrepancies contribute to the $I_{\text{obs, wrong intensity}}$ term with reduced weight. The penalty terms account for simulated peaks that are missing from the observed pattern ($I_{\text{sim, missing}}$) and for extra simulated peaks not supported by the experimental data ($I_{\text{sim, extra}}$), with stronger penalties assigned to extra peaks to discourage over-parameterized phase combinations.

This peak-match score is intended as a fast, physically motivated heuristic to prioritize promising candidate interpretations during the tree-based search, rather than as a

replacement for full Rietveld refinement. As such, it is used for candidate pruning and ranking prior to computing final posterior probabilities.

11.3 Trustworthiness flag

We convert multiple quality metrics associated with an interpretation into a single trustworthiness score used to flag potentially unreliable results for manual review. The trustworthiness score combines both data-driven and chemistry-based diagnostics, each mapped to a continuous value in the range $[0, 1]$, and is designed to reflect how strongly an interpretation satisfies a set of physically motivated criteria.

Metrics that are natively bounded—specifically the composition-balance score and the LLM-derived likelihood—are already defined on the $[0, 1]$ interval and therefore require no additional normalization. The remaining metrics are normalized as follows. The refined R_{wp} value is mapped to $[0, 1]$ using a clipped linear normalization, with values below zero mapped to 1 and values above a maximum cutoff of $R_{\mathrm{wp}} = 60$ mapped to 0, ensuring robustness to extreme outliers. The peak-match score is normalized using a sigmoid function with slope parameter $k = 3$ and center $c = 0.3$, chosen to emphasize meaningful peak agreement while smoothly down-weighting poorer matches.

For trustworthiness evaluation, each normalized metric is converted into a diagnostic confidence score using a sigmoid centered at a metric-specific threshold (Table 1), such that values comfortably satisfying the threshold yield scores close to 1, while values violating the threshold yield scores near 0. This smooth transition ($p = 0.5$ at the threshold) avoids hard cutoffs and captures the degree to which each criterion supports the interpretation.

For each diagnostic, the deviation from its threshold is interpreted as a soft penalty bounded between 0 and 1. The overall trustworthiness score is then computed as one minus the average penalty across all included diagnostics, ensuring that multiple moderate violations can jointly indicate low confidence while preventing any single metric from dominating.

The threshold criteria for the entries in Table 1 were selected based on commonly used values within our group and refined through qualitative tuning on the TRI dataset. Scale parameters were set to reflect the natural dynamic range of each diagnostic: intensity-based metrics (signal-above-background, background-overshoot) use large scale values (2000, 300) consistent with their typical range of thousands of intensity units; the signal-to-background ratio uses scale 3.0 consistent with its typical range of 0–50; bounded metrics on $[0, 1]$ (LLM likelihood, balance score, peak-matching score) use scale 0.10; and R_{wp} uses scale 5.0 reflecting its typical operating range of 5–60. A global temperature parameter $T = 1.25$ is applied in logit space to scale the sharpness of all sigmoid transitions: values $T > 1$ soften the transitions (making penalties more gradual), while $T < 1$ sharpens them. The value $T = 1.25$ was chosen to avoid overly abrupt penalties while maintaining sensitivity to diagnostic violations. All diagnostic weights are set to $w_k = 1.0$, reflecting equal contribution of each diagnostic. The overall threshold value of 0.60 was chosen based on F1 analysis on the 20-sample test set, using majority chemist trust labels as ground truth. A sensitivity analysis over thresholds from 0.40 to 0.80 (Figure S2) shows that F1 remains stable at 0.897 across thresholds from 0.40 to 0.60, before dropping sharply to 0.696 at 0.65. The threshold of 0.60 was therefore selected as the most conservative value within the stable plateau. Interpretations with a combined trustworthiness score below 0.60 are considered unreliable and automatically flagged for

further inspection.

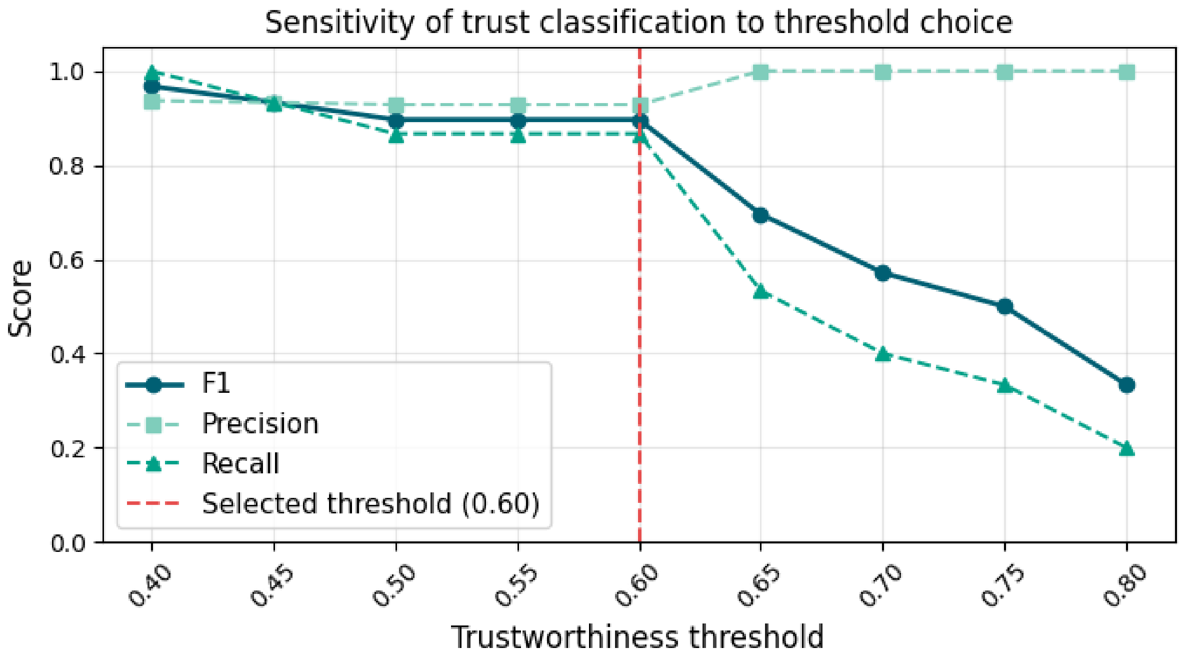

Figure S2: Sensitivity of trust classification F1 score to threshold choice, computed on the 20-sample test set using majority chemist trust labels as ground truth. F1 remains stable at 0.897 for thresholds between 0.40 and 0.60, before dropping sharply at higher values. The selected threshold of 0.60 (red dashed line) lies at the conservative edge of the stable region.

Below we define the individual signal- and background-based metrics used in the trustworthiness calculation.

Signal-above-background score (area per degree 2θ).

$$S = \frac{1}{\Delta 2\theta} \sum_{i=1}^{N} \left[\max\left(I_{\mathrm{obs},i}, I_{\mathrm{bkg},i}\right) - I_{\mathrm{bkg},i} \right]$$

A larger S means the peaks stand well above the background.

Background-overshoot penalty (area per degree 2θ).

$$B = \frac{1}{\Delta 2\theta} \sum_{i=1}^{N} \max\left(I_{\mathrm{bkg},i} - I_{\mathrm{obs},i},\ 0\right)$$

This penalizes any portion of the fitted background that rises above the experimental signal.

Choice of 2θ window: $[10^\circ, 70^\circ]$.

12 Chemist evaluations

12.1 Multi-project evaluation

Details of all 20 samples in the expert-reviewed test set—including their synthesis conditions, the phases proposed by the lowest-Rwp and AIF interpretations, and the per-chemist selections for each sample—are provided in Table S2.

**Sample ID**	**Target**	**Precursors**	**Synthesis conditions**	**Lowest Rwp**	**AIF**	**Chemist A**	**Chemist B**	**Chemist C**	**Chemist D**
TRI_80	CaV_2O_3	[$CaCO_3$, V_2O_3]	TF-Ar, 500°C, 12 h	[V_2O_3_15, $CaCO_3$_167]	[V_2O_3_167, $CaCO_3$_167]	AIF	AIF	both	AIF
TRI_81	$LiVO_2$	[Li_2CO_3, V_2O_5]	TF-Ar, 400°C, 12 h	[$Li_{1.94}V_6O_{16}$_11, Li_4CO_5_15]	[LiV_3O_8_11]	Lowest Rwp	AIF	Lowest Rwp	AIF
TRI_90	CaV_2O_3	[$CaCO_3$, V_2O_3]	TF-Ar, 700°C, 12 h	[V_2O_3_15, $Ca_5V_3O_{13}$_176]	[V_2O_3_167, $Ca_5V_3O_{13}$_176]	AIF	AIF	AIF	neither
TRI_106	$NaVO_2$	[Na_2CO_3, V_2O_5, C]	TF-Ar, 700°C, 12 h	[V_2O_3_15, CO_194]	[V_2O_3_167]	neither	neither	neither	neither
TRI_114	$LiVO_2$	[Li_2CO_3, V_2O_5]	TF-Ar + H_2, 600°C, 12 h	[Li_3VO_4_31, V_2O_3_15, V_4O_7_2]	[Li_3VO_4_31, V_2O_3_167, V_4O_7_2]	AIF	AIF	both	AIF
TRI_183	$MgVO_3$	[$MgCO_3$, V_2O_5]	BF, 500°C, 12 h	[$Mg_2V_2O_7$_2, MgV_2O_6_12, VO_2_58]	[$Mg_2V_2O_7$_2, MgV_2O_6_12]	neither	neither	both	AIF
PG_1048	$VCrO_4$	[Cr_2O_3,V_2O_5]	BF, 400°C, 1 h	[$V_4Cr_{0.22}O_{10.32}$_59, $V_{3.6}Cr_{8.4}O_{18}$_167, Cr_2O_3_167, V_2O_5_59]	[V_2O_5_59, $V_{.63}Cr_{8.4}O_{18}$_167, Cr_2O_3_167]	both	both	both	AIF
PG_0849	$CoWO_4$	[CoO, WO_3]	BF, 900°C, 1 h	[WO_3_2, $CoWO_4$_13]	[WO_3_14, $CoWO_4$_13]	both	AIF	both	AIF
ARR_46	$CaTiNiP_2O_9$	[CaO, $NH_4H_2PO_4$, NiO, TiO_2]	BF, 900°C, 4 h	[$CaP_2(HO_2)_4$_2, $Ti_{7.3336}O_{16}$_61, $CaTi_4(PO_4)_6$_148]	[$Ti_{7.336}O_{16}$_61, $CaTi_4(PO_4)_6$_148]	neither	neither	neither	neither
TRI_28	$CaVO_2$	[$CaCO_3$, V_2O_3]	BF, 700°C, 8 h	[$Ca_2V_2O_7$_2, CaC_2_15]	[$Ca_2V_2O_7$_2, CaV_2O_6_12]	AIF	neither	AIF	AIF
TRI_104	CaV_2O_3	[$CaCO_3$, V_2O_5, C]	TF-Ar, 700°C, 12 h	[V_3O_5_15, V_2O_3_167, V_6O_{13}_69, V_5O_9_2]	[V_3O_5_15, V_2O_3_167, V_6O_{13}_12, $Ca_5V_3O_{13}$_176, CaO_225]	neither	neither	neither	neither
TRI_111	$NaVO_2$	[Na_2CO_3, V_2O_5]	TF-Ar + H_2, 600°C, 12 h	[NaV_2O_4_59, V_2O_3_167, $NaVO_2$_166, C_61, C_15]	[NaV_2O_4_59, V_2O_3_167, $NaVO_2$_166, $V_{14}O_{6.16}$_12]	AIF	neither	both	AIF
TRI_113	KVO_2	[K_2CO_3, V_2O_5]	TF-Ar + H_2, 600°C, 12 h	[KVO_3_57, K_3VO_4_121, C_194]	[KVO_3_57, K_3VO_4_121]	neither	neither	neither	neither
ARR_45	$CaTiNiP_2O_9$	[CaO, $(NH_4)_2HPO_4$, $Ni(OH)_2$, TiO_2]	BF, 900°C, 4 h	[$TiNiO_3$_148]	[$Ti_{11.396}N_4O_{16}$_63, $CaP_2(HO_2)_4$_2, Ti_3O_5_12]	neither	neither	neither	neither
ARR_54	$MgCuP_2O_7$	[CuO, $MgCO_3$, $NH_4H_2PO_4$]	BF, 700°C, 4 h	[$Mg_2P_2O_7$_14, CuO_15, Cu_3P_164]	[$Mg_2P_2O_7$_14, CuO_15]	neither	AIF	both	neither
ARR_34	$MnAgO_2$	[Ag_2CO_3, Mn_2O_3]	BF, 400°C, 4 h	[Ag_225, Mn_2O_3_61, Mn0.6Ag3.3_225]	[Ag_225, Mn_2O_3_206]	AIF	AIF	AIF	both
ARR_208	Ta_4PbO_{11}	[PbO_2, Ta_2O_5]	BF, 700°C, 4 h	[Ta_2O_5_25, $Ta_{16}Pb_{12}O_{50}$_227]	[Ta_2O_5_25, Ta_2PbO_7_227]	both	both	both	AIF
TRI-102	CaV_2O_3	[$CaCO_3$, V_2O_3]	TF-Ar + H_2, 700°C, 12 h	[V_2O_3_15, CaO_225, $Ca_5V_3O_{13}$_176, $CaVO_3$_62]	[V_2O_3_167, CaO_225, $Ca_5V_3O_{13}$_176, $CaVO_3$_62]	both	AIF	both	AIF
ARR_110	$BaMn_8O_{16}$	[BaO_2, MnO]	BF, 600°C, 4 h	[Mn_2O_3_61, $BaMnO_3$_185, MnO_2_136]	[Mn_2O_3_61, $BaMnO_3$_194, MnO_2_136]	neither	neither	both	both
ARR_194	$Y_3In_2Ga_3O_{12}$	[Y_2O_3, Ga_2O_3, In_2O_3]	BF, 800°C, 4 h	[Y_2O_3_206, In28Ga3.5O48_206, Ga_2O_3_12]	[Y_2O_3_206, In_2O_3_206, Ga_2O_3_12]	both	neither	both	AIF

Table S2: Comparison of interpretation assignments between lowest-R_{wp} and AIF selections, with Chemist A–D preferences.

In addition to the strict agreement metric reported —where two chemists are considered to agree only when they select exactly the same category (AIF, Lowest-R_{wp}, Both, or Neither)—we also computed a more permissive partial agreement.

In this relaxed definition, the category Both is treated as compatible with either AIF or lowest-R_{wp}. For example, if one chemist selects AIF and another selects Both, the pair is counted as being in agreement. Similarly, Both is treated as compatible with lowest-R_{wp}.

Figure S3 reports the resulting strict inter-chemist agreement matrix. Summed strict pairwise agreement values are A: 185%, B: 160%, C: 150%, and D: 135%.

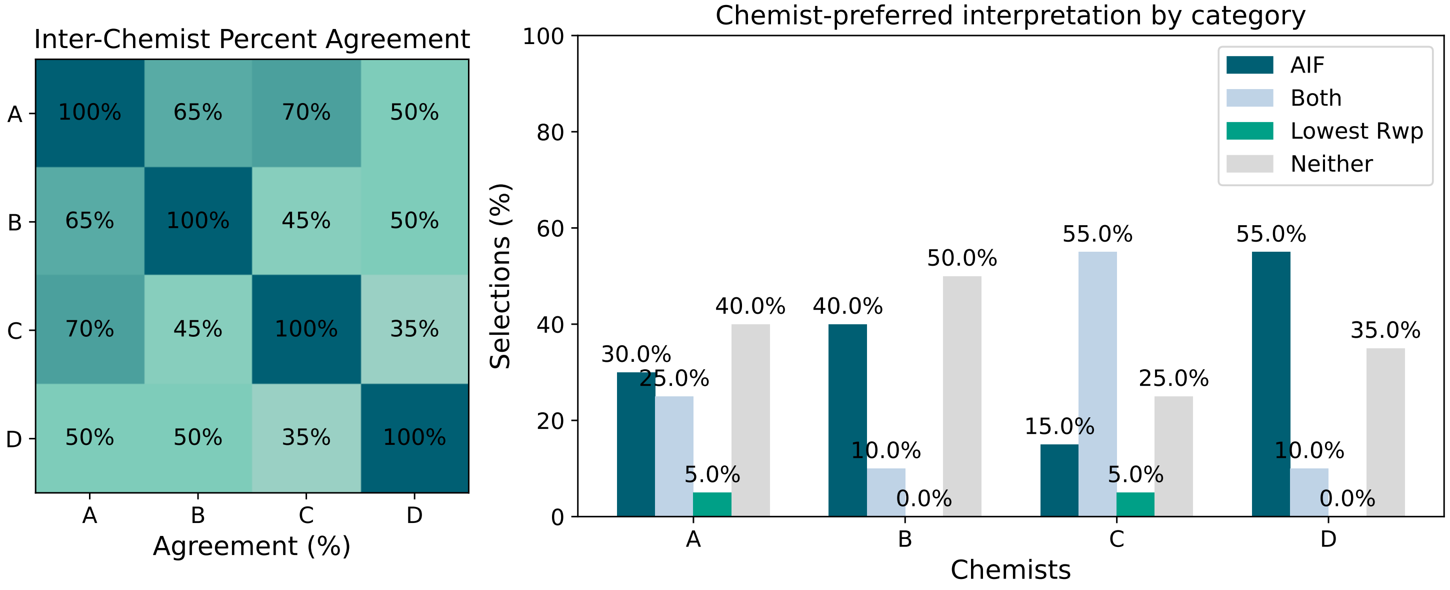

Figure S3: Inter-chemist agreement and reviewer selection patterns for the 20-sample benchmark dataset. The heat map in the left panel shows strict pairwise agreement percentages between chemists, where agreement requires selecting exactly the same category. The bar charts in the right panel show the distribution of selections for each of the four chemists individually.

Figure S4 reports the resulting partial agreement matrix. Agreement values increase relative to the strict definition, with pairwise agreement ranging from 65% to 85%.

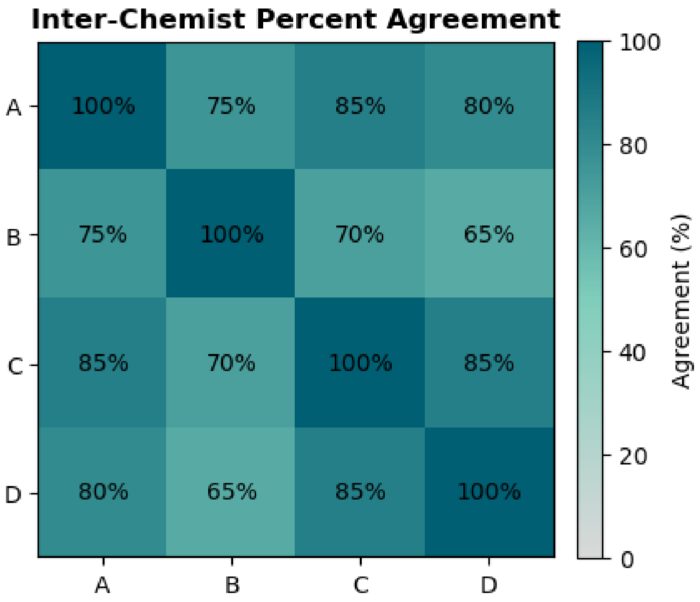

Figure S4: Inter-chemist percent agreement across the 20-sample benchmark. Each cell reports the fraction of samples for which two chemists selected the same interpretation category (AIF, lowest-Rwp, both, or neither).

12.1.1 Calibration of interpretation-level metric weights

The relative weights used to compute interpretation-level probabilities were calibrated using a coarse grid search over candidate weight combinations. The calibration was performed using a set of 30 expert-reviewed samples. For each sample, the expert examined the full set of candidate interpretations and either confirmed the default AIF-selected interpretation or selected an alternative interpretation judged to be more physically and crystallographically plausible.

A grid search was conducted over weights in the range $[0, 1]$ with a step size of 0.1 for each of the four metrics. For each candidate weight set, interpretation probabilities were recomputed for all interpretations associated with each sample, and the highest-probability interpretation was identified. Weight sets were scored based on whether the interpretation with the highest recomputed probability matched the expert-selected interpretation.

The objective of this procedure was to identify a robust weighting scheme that assigns higher probability to expert-preferred interpretations when discrepancies arise, while preserving stable interpretation rankings for samples where the default AIF selection was confirmed. The optimized weights reported in the main text were selected from among the highest-scoring combinations and used consistently throughout subsequent analyses.

Of 14,400 valid weight combinations explored (11^4 = 14,641 minus 241 excluded for zero denominators in Equations 2 and 3), 13 tied at the maximum training score of 159 (Figure S5). The reported weights were selected from among these as the combination most consistent with established diffraction practice ($w_{R_{\mathrm{wp}}} = 1.0$ as the primary figure of merit).

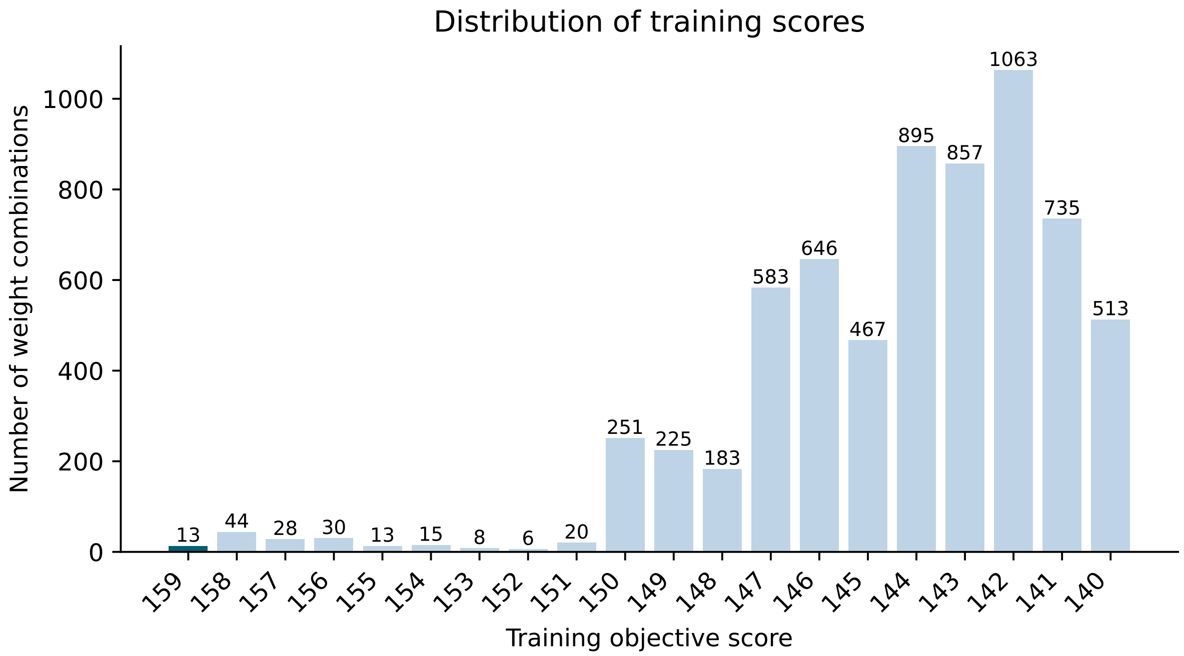

Figure S5: Distribution of training objective scores across 14,400 valid weight combinations.

12.1.2 Trust alignment between AIF and chemists

We next evaluated whether AIF assigns high confidence to the same interpretations trusted by human experts. Figure S6 provides trust matrices for each chemist individually. All four reviewers exhibit similar alignment trends with AIF, though with varying degrees of strictness. Chemists D and C show the strongest agreement with AIF, while A and B are more conservative yet still exhibit consistent patterns. These results confirm that AIF's confidence calibration is robust across reviewers and is not driven by a single evaluator. These lead to an overall 56.2% of samples, both AIF and chemists jointly trusted the same interpretation, 13.8%, chemists trusted an interpretation that AIF deemed low-confidence; the reverse occurred in 6.2% of samples. Mutual non-trust occurred in 23.8% of samples. These results highlight that AIF's internal confidence scores meaningfully align with human judgment.

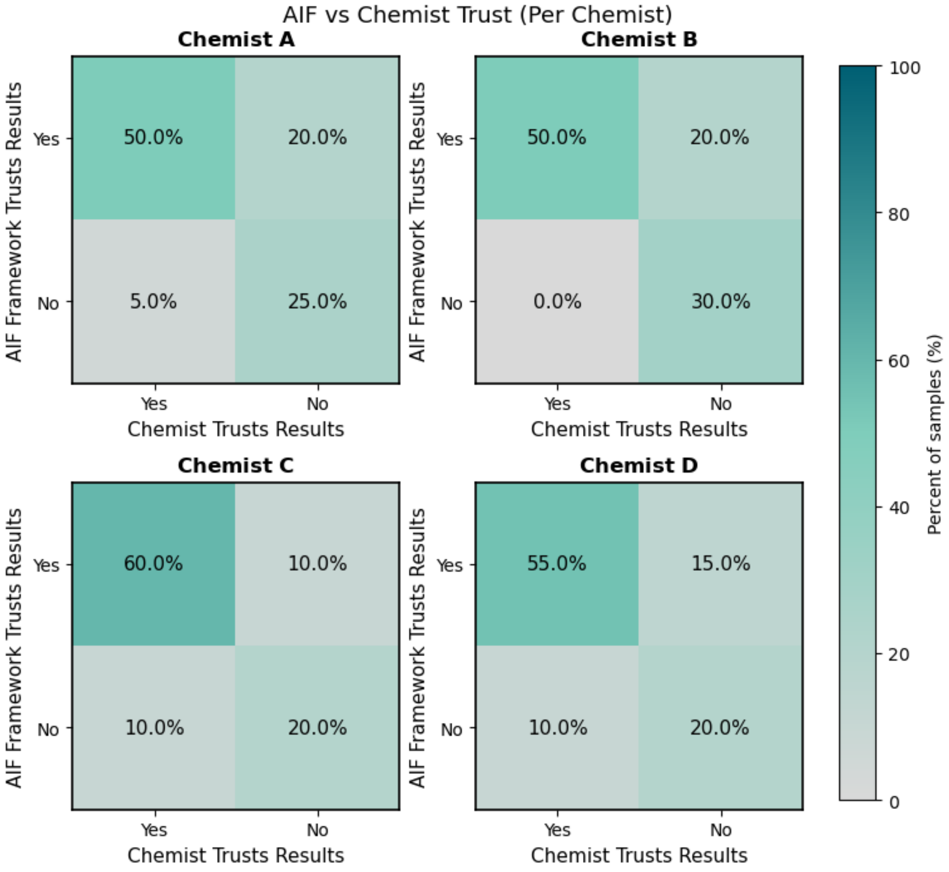

Figure S6: Trust alignment between AIF and each chemist individually. For each reviewer, entries show the percentage of samples for which AIF and the chemist either jointly trusted, jointly rejected, or disagreed on the trustworthiness of the interpretation. While the strictness of chemists varies, all four display consistent trends and substantial overlap with AIF confidence judgments.

12.1.3 Analysis of Lowest-R_{wp} Preferred Cases

Finally, we examined the rare instances in which chemists preferred the lowest-R_{wp} refinement over the AIF interpretation. Across the 20 samples, only a single case (TRI-81) had at least two chemists selecting the lowest-R_{wp} solution as the more credible interpretation. This sample targeted $LiVO_2$ and was synthesized from Li_2CO_3 and V_2O_5 in a tube furnace under flowing Ar at 400 °C for 12 h.

Figure S7 shows the corresponding diffraction pattern. In this sample, the lowest-R_{wp} refinement reproduced the experimental profile with fewer missing peaks than the AIF-selected interpretation. This more complete peak coverage likely drove the chemists' preference, even though both interpretations were similar and chemically plausible.

Although the lowest-R_{wp} refinement achieved slightly stronger purely data-driven diagnostics—a marginally higher composition-balance score (0.98 vs. 0.94) and one fewer missing peaks—AIF did not adopt it because the chemically informed prior penalized the additional carbonate phase present in that interpretation. The LLM assigned a higher interpretation likelihood to the AIF-selected solution ($\ell = 0.75$) and explicitly framed it as chemically plausible:

> *"This interpretation is plausible as it includes a likely phase, LiV3O8, which*

matches the synthesis conditions. However, the absence of other phases or unreacted precursors slightly reduces the likelihood, as a single-phase product is less common in solid-state synthesis."

In contrast, the lowest-R_{wp} solution received a lower likelihood ($\ell = 0.60$) because it invoked an unlikely carbonate product:

"While LiV3O8 is a plausible phase, the presence of Li4CO5 significantly reduces the credibility of this interpretation. The composition balance score is high, but the inclusion of an unlikely carbonate phase suggests misidentification or incomplete reaction."

Thus, despite a modest advantage in fit/balance metrics, the lowest-R_{wp} interpretation is downweighted by AIF due to the chemically implausible phase assignment, shifting preference toward the AIF interpretation.

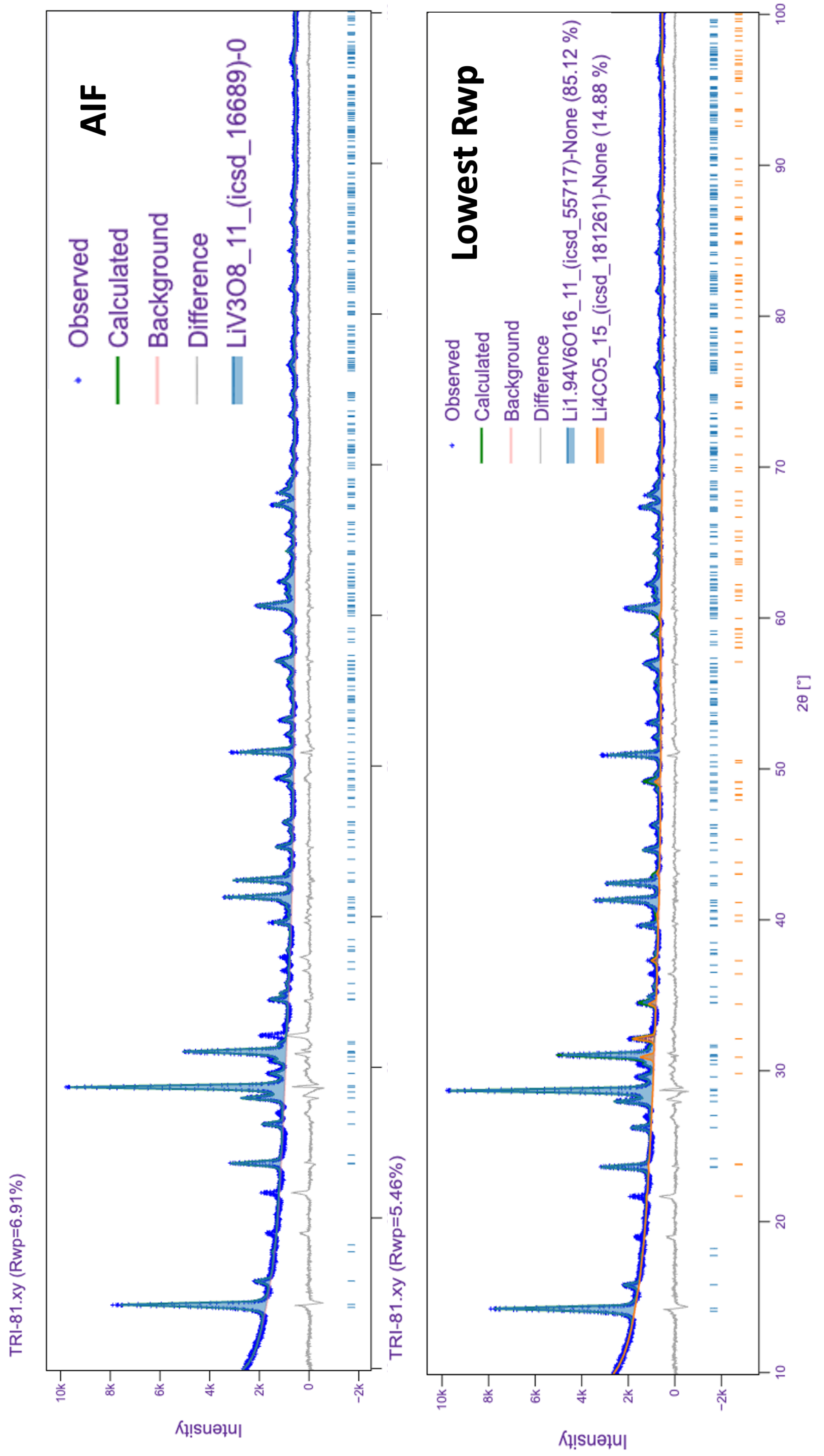

Figure S7: Diffraction pattern for sample TRI-81, the only case in which two chemists preferred the lowest-R_{wp} refinement over the AIF interpretation.

12.2 Precursor Genome evaluation

A detailed summary of the Genome dataset samples—including their synthesis conditions, the chemists' assessments of the lowest-Rwp and AIF interpretations, and whether the chemists judged the AIF result to be better, worse, or equivalent to the lowest-Rwp solution—is provided in Table S3.

Sample ID	Target	Precursors	Synthesis conditions	Agreement with Lowest-Rwp	Agreement with AIF	AIF vs. Lowest Rwp
PG_1	$Fe_2Ag_2H_8C_4O_{13}$	Ag_2O, $FeC_2O_4(H_2O)_2$	BF, 200°C, 1 h	TRUE	TRUE	Same
PG_3	$Mn_6Ag_6O_{11}$	Ag_2O, Mn_3O_4	BF, 200°C, 1 h	TRUE	TRUE	AIF is better
PG_4	$MnAgO_2$	Ag_2O, Mn_2O_3	BF, 200°C, 1 h	TRUE	TRUE	AIF is better
PG_7	$Si_2Ag_2O_5$	Ag_2O, SiO_2	BF, 200°C, 1 h	TRUE	TRUE	AIF is better
PG_8	$Ag_2Sn_2O_5$	$FeH_4(CO_3)_2$, SnO_2, $FeSnH_4(CO_4)_2$	BF, 200°C, 1 h	TRUE	FALSE	Same
PG_14	$Al_3Co_3H_9O_{13}$	$Al(OH)_3$, Co_3O_4	BF, 200°C, 1 h	TRUE	TRUE	AIF is better
PG_16	$Al_2Ga_2(H_2O_3)_3$	$Al(OH)_3$, Ga_2O_3	BF, 200°C, 1 h	TRUE	TRUE	Same
PG_21	$AlNiH_3O_4$	$Al(OH)_3$, NiO	BF, 200°C, 1 h	TRUE	TRUE	Same
PG_31	$CoNiO_2$	CoO, NiO	BF, 1100°C, 1 h	TRUE	TRUE	Same
PG_37	$Mn_3Fe_3O_8$	Fe_3O_4, Mn_3O_4	BF, 1000°C, 1 h	TRUE	TRUE	Same
PG_39	$Ga_2Ni_2O_5$	Ga_2O_3, NiO	BF, 1100°C, 1 h	TRUE	TRUE	Same
PG_41	$Co_6Ag_6O_{11}$	Ag_2O, Co_3O_4	BF, 200°C, 1 h	FALSE	FALSE	Same
PG_42	$CrAgO_2$	Ag_2O, Cr_2O_3	BF, 200°C, 1 h	FALSE	FALSE	Same
PG_43	$Ag_2Ge_2O_5$	Ag_2O, GeO_2	BF, 200°C, 1 h	FALSE	FALSE	Same
PG_44	$K_4Ag_4H_6C_2O_{11}$	Ag_2O, $K_2CO_3(H_2O)_{1.5}$	BF, 200°C, 1 h	FALSE	TRUE	Same
PG_45	$Mn_2Ag_2O_5$	Ag_2O, MnO_2	BF, 200°C, 1 h	FALSE	TRUE	Same
PG_46	$Na_2Ag_2CO_4$	Ag_2O, Na_2CO_3	BF, 200°C, 1 h	FALSE	TRUE	AIF is better
PG_47	$BaMoCO_6$	$BaCO_3$, MoO_3	BF, 400°C, 1 h	FALSE	TRUE	AIF is better
PG_48	$BaPH_9CN_2O_7$	$BaCO_3$, $(NH_4)_2HPO_4$	BF, 200°C, 1 h	FALSE	FALSE	AIF is better
PG_50	$BaFeH_4(CO_4)_2$	BaO_2, $FeC_2O_4(H_2O)_2$	BF, 200°C, 1 h	FALSE	FALSE	Same
PG_51	$BaPH_6NO_6$	BaO_2, $NH_4H_2PO_4$	BF, 200°C, 1 h	FALSE	FALSE	Same
PG_53	$CaPH_6CNO_7$	$CaCO_3$, $NH_4H_2PO_4$	BF, 200°C, 1 h	FALSE	TRUE	AIF is better
PG_54	$CaPbCO_4$	$CaCO_3$, PbO	BF, 500°C, 1 h	FALSE	FALSE	Same
PG_55	$CoMoO_4$	CoO, MoO_3	BF, 400°C, 1 h	FALSE	FALSE	Same
PG_57	$Co_3Mo_3O_{13}$	Co_3O_4, MoO_3	BF, 400°C, 1 h	FALSE	FALSE	Same
PG_60	$CuNiO_2$	CuO, NiO	BF, 800°C, 1 h	FALSE	TRUE	AIF is better
PG_61	$Sr_3Fe_3C_3O_{13}$	Fe_3O_4, $SrCO_3$	BF, 900°C, 1 h	FALSE	FALSE	Same
PG_63	$MgFeH_4C_2O_7$	$FeC_2O_4(H_2O)_2$, MgO	BF, 200°C, 1 h	FALSE	FALSE	Same
PG_64	$MnFeH_4C_2O_7$	$FeC_2O_4(H_2O)_2$, MnO	BF, 200°C, 1 h	FALSE	FALSE	Same
PG_65	$FeSnH_4(CO_4)_2$	$FeC_2O_4(H_2O)_2$, SnO_2	BF, 200°C, 1 h	FALSE	FALSE	Same
PG_66	$Sr_2Ga_2C_2O_9$	Ga_2O_3, $SrCO_3$	BF, 900°C, 1 h	FALSE	FALSE	Same
PG_69	$MgSiO_3$	MgO, SiO_2	BF, 1000°C, 1 h	FALSE	TRUE	Same
PG_73	$MnSiO_3$	MnO, SiO_2	BF, 1000°C, 1 h	FALSE	FALSE	Same
PG_78	$MnPbO_3$	MnO_2, PbO	BF, 300°C, 1 h	FALSE	TRUE	AIF is better

Table S3: Chemist evaluations comparing lowest-R_{wp} and AIF suggestions on the Genome dataset (34 re-evaluated samples). Numbering follows Tables S4 and S5.

Sample ID	Target	Precursors	Synthesis conditions	Agreement with Lowest-R_{wp}	AIF and Lowest-R_{wp} agree
PG_1	$Fe_2Ag_2H_8C_4O_{13}$	Ag_2O, $FeC_2O_4(H_2O)_2$	BF, 200°C, 1 h	TRUE	No
PG_2	$GaAgO_2$	Ag_2O, Ga_2O_3	BF, 200°C, 1 h	TRUE	Yes
PG_3	$Mn_6Ag_6O_{11}$	Ag_2O, Mn_3O_4	BF, 200°C, 1 h	TRUE	No
PG_4	$MnAgO_2$	Ag_2O, Mn_2O_3	BF, 200°C, 1 h	TRUE	No
PG_5	$NbAgO_3$	Ag_2O, Nb_2O_5	BF, 200°C, 1 h	TRUE	Yes
PG_6	$AgSbO_2$	Ag_2O, Sb_2O_3	BF, 200°C, 1 h	TRUE	Yes
PG_7	$Si_2Ag_2O_5$	Ag_2O, SiO_2	BF, 200°C, 1 h	TRUE	No
PG_8	$Ag_2Sn_2O_5$	Ag_2O, SnO_2	BF, 200°C, 1 h	TRUE	No
PG_9	$Sr_2Ag_2C_2O_7$	Ag_2O, $SrCO_3$	BF, 200°C, 1 h	TRUE	Yes
PG_10	$Ti_2Ag_2O_5$	Ag_2O, TiO_2	BF, 200°C, 1 h	TRUE	Yes
PG_11	$BaAlH_3CO_6$	$Al(OH)_3$, $BaCO_3$	BF, 200°C, 1 h	TRUE	Yes
PG_12	$BaAlH_3O_5$	$Al(OH)_3$, BaO_2	BF, 200°C, 1 h	TRUE	Yes
PG_13	$Al_2Bi_2(H_2O_3)_3$	$Al(OH)_3$, Bi_2O_3	BF, 200°C, 1 h	TRUE	Yes
PG_14	$Al_3Co_3H_9O_{13}$	$Al(OH)_3$, Co_3O_4	BF, 200°C, 1 h	TRUE	No
PG_15	$Al_2Cr_2(H_2O_3)_3$	$Al(OH)_3$, Cr_2O_3	BF, 200°C, 1 h	TRUE	Yes
PG_16	$Al_2Ga_2(H_2O_3)_3$	$Al(OH)_3$, Ga_2O_3	BF, 200°C, 1 h	TRUE	No
PG_17	$AlGeH_3O_5$	$Al(OH)_3$, GeO_2	BF, 200°C, 1 h	TRUE	Yes
PG_18	$MgAlH_3O_4$	MgO, $Al(OH)_3$	BF, 200°C, 1 h	TRUE	Yes
PG_19	$MnAlH_3O_4$	$Al(OH)_3$, MnO	BF, 200°C, 1 h	TRUE	Yes
PG_20	$AlMo(HO_2)_3$	$Al(OH)_3$, MoO_3	BF, 200°C, 1 h	TRUE	Yes
PG_21	$AlNiH_3O_4$	$Al(OH)_3$, NiO	BF, 200°C, 1 h	TRUE	No
PG_22	AlH_3PbO_4	$Al(OH)_3$, PbO	BF, 200°C, 1 h	TRUE	Yes
PG_23	$AlSiH_3O_5$	$Al(OH)_3$, SiO_2	BF, 200°C, 1 h	TRUE	Yes
PG_24	$TiAlH_3O_5$	$Al(OH)_3$, TiO_2	BF, 200°C, 1 h	TRUE	Yes
PG_25	$MgBH_3O_4$	$B(OH)_3$, MgO	BF, 200°C, 1 h	TRUE	Yes
PG_26	Ba_2CO_5	$BaCO_3$, BaO_2	BF, 200°C, 1 h	TRUE	Yes
PG_27	$BaCoCO_4$	$BaCO_3$, CoO	BF, 400°C, 1 h	TRUE	Yes
PG_28	$MgCoO_2$	CoO, MgO	BF, 1100°C, 1 h	TRUE	Yes
PG_29	$MnCoO_2$	CoO, MnO	BF, 1100°C, 1 h	TRUE	Yes
PG_30	$Mn_3Co_3O_7$	CoO, Mn_3O_4	BF, 1000°C, 1 h	TRUE	Yes
PG_31	$CoNiO_2$	CoO, NiO	BF, 1100°C, 1 h	TRUE	No
PG_32	$Y_2Co_2O_5$	CoO, Y_2O_3	BF, 1100°C, 1 h	TRUE	Yes
PG_33	$Mg_2Cr_2O_5$	Cr_2O_3, MgO	BF, 1100°C, 1 h	TRUE	Yes
PG_34	$NbCrO_4$	Cr_2O_3, Nb_2O_5	BF, 900°C, 1 h	TRUE	Yes
PG_35	$Sr_2Cr_2C_2O_9$	Cr_2O_3, $SrCO_3$	BF, 900°C, 1 h	TRUE	Yes
PG_36	$YCrO_3$	Cr_2O_3, Y_2O_3	BF, 1100°C, 1 h	TRUE	Yes
PG_37	$Mn_3Fe_3O_8$	Fe_3O_4, Mn_3O_4	BF, 1000°C, 1 h	TRUE	No
PG_38	$NbFeO_4$	Fe_2O_3, Nb_2O_5	BF, 900°C, 1 h	TRUE	Yes
PG_39	$Ga_2Ni_2O_5$	Ga_2O_3, NiO	BF, 1100°C, 1 h	TRUE	No
PG_40	$VInO_3$	In_2O_3, V_2O_3	BF, 1100°C, 1 h	TRUE	Yes

Table S4: The 40 samples for which the chemist agreed with the lowest-R_{wp} interpretation (TRUE cohort). The final column indicates whether the AIF selection matched the lowest-R_{wp} baseline.

Sample ID	Target	Precursors	Synthesis conditions	Agreement with Lowest-R_{wp}	AIF and Lowest-R_{wp} agree
PG_41	$Co_6Ag_6O_{11}$	Ag_2O, Co_3O_4	BF, 200°C, 1 h	FALSE	No
PG_42	$CrAgO_2$	Ag_2O, Cr_2O_3	BF, 200°C, 1 h	FALSE	No
PG_43	$Ag_2Ge_2O_5$	Ag_2O, GeO_2	BF, 200°C, 1 h	FALSE	No
PG_44	$K_4Ag_4H_6C_2O_{11}$	Ag_2O, $K_2CO_3(H_2O)_{1.5}$	BF, 200°C, 1 h	FALSE	No
PG_45	$Mn_2Ag_2O_5$	Ag_2O, MnO_2	BF, 200°C, 1 h	FALSE	No
PG_46	$Na_2Ag_2CO_4$	Ag_2O, Na_2CO_3	BF, 200°C, 1 h	FALSE	No
PG_47	$BaMoCO_6$	$BaCO_3$, MoO_3	BF, 400°C, 1 h	FALSE	No
PG_48	$BaPH_9CN_2O_7$	$BaCO_3$, $(NH_4)_2HPO_4$	BF, 200°C, 1 h	FALSE	No
PG_49	$BaCuO_3$	BaO_2, CuO	BF, 200°C, 1 h	FALSE	Yes
PG_50	$BaFeH_4(CO_4)_2$	BaO_2, $FeC_2O_4(H_2O)_2$	BF, 200°C, 1 h	FALSE	No
PG_51	$BaPH_6NO_6$	BaO_2, $NH_4H_2PO_4$	BF, 200°C, 1 h	FALSE	No
PG_52	$CaFeH_4(CO_3)_3$	$CaCO_3$, $FeC_2O_4(H_2O)_2$	BF, 200°C, 1 h	FALSE	Yes
PG_53	$CaPH_6CNO_7$	$CaCO_3$, $NH_4H_2PO_4$	BF, 200°C, 1 h	FALSE	No
PG_54	$CaPbCO_4$	$CaCO_3$, PbO	BF, 500°C, 1 h	FALSE	No
PG_55	$CoMoO_4$	CoO, MoO_3	BF, 400°C, 1 h	FALSE	No
PG_56	$Nb_2Co_2O_7$	CoO, Nb_2O_5	BF, 900°C, 1 h	FALSE	Yes
PG_57	$Co_3Mo_3O_{13}$	Co_3O_4, MoO_3	BF, 400°C, 1 h	FALSE	No
PG_58	$Na_2Cr_2CO_6$	Cr_2O_3, Na_2CO_3	BF, 500°C, 1 h	FALSE	Yes
PG_59	$CrSbO_3$	Cr_2O_3, Sb_2O_3	BF, 300°C, 1 h	FALSE	Yes
PG_60	$CuNiO_2$	CuO, NiO	BF, 800°C, 1 h	FALSE	No
PG_61	$Sr_3Fe_3C_3O_{13}$	Fe_3O_4, $SrCO_3$	BF, 900°C, 1 h	FALSE	No
PG_62	$Fe_2Pb_2O_5$	Fe_2O_3, PbO	BF, 500°C, 1 h	FALSE	Yes
PG_63	$MgFeH_4C_2O_7$	$FeC_2O_4(H_2O)_2$, MgO	BF, 200°C, 1 h	FALSE	No
PG_64	$MnFeH_4C_2O_7$	$FeC_2O_4(H_2O)_2$, MnO	BF, 200°C, 1 h	FALSE	No
PG_65	$FeSnH_4(CO_4)_2$	$FeC_2O_4(H_2O)_2$, SnO_2	BF, 200°C, 1 h	FALSE	No
PG_66	$Sr_2Ga_2C_2O_9$	Ga_2O_3, $SrCO_3$	BF, 900°C, 1 h	FALSE	No
PG_67	$Ti_2In_2O_7$	In_2O_3, TiO_2	BF, 1100°C, 1 h	FALSE	Yes
PG_68	$Mg_3Mn_3O_7$	MgO, Mn_3O_4	BF, 1000°C, 1 h	FALSE	Yes
PG_69	$MgSiO_3$	MgO, SiO_2	BF, 1000°C, 1 h	FALSE	No
PG_70	$MgSnO_3$	MgO, SnO_2	BF, 1000°C, 1 h	FALSE	Yes
PG_71	$Mg_2V_2O_5$	MgO, V_2O_3	BF, 1100°C, 1 h	FALSE	Yes
PG_72	$MnPbO_2$	MnO, PbO	BF, 500°C, 1 h	FALSE	Yes
PG_73	$MnSiO_3$	MnO, SiO_2	BF, 1000°C, 1 h	FALSE	No
PG_74	$Mn_3Sn_3O_{10}$	Mn_3O_4, SnO_2	BF, 1000°C, 1 h	FALSE	Yes
PG_75	$Mn_3Zn_3O_7$	ZnO, Mn_3O_4	BF, 1000°C, 1 h	FALSE	Yes
PG_76	Mn_4O_5	Mn_2O_3, MnO	BF, 500°C, 1 h	FALSE	Yes
PG_77	$MnSbO_3$	Mn_2O_3, Sb_2O_3	BF, 300°C, 1 h	FALSE	Yes
PG_78	$MnPbO_3$	MnO_2, PbO	BF, 300°C, 1 h	FALSE	No
PG_79	$Mn_2Sb_2O_7$	MnO_2, Sb_2O_3	BF, 300°C, 1 h	FALSE	Yes
PG_80	$Zn_2Sb_2O_5$	Sb_2O_3, ZnO	BF, 300°C, 1 h	FALSE	Yes

Table S5: The 40 samples for which the chemist disagreed with the lowest-R_{wp} interpretation (FALSE cohort). The final column indicates whether the AIF selection matched the lowest-R_{wp} baseline.

12.3 Calibration and predictive value of AIF confidence (ROC curves and Brier score)

To gauge whether AIF's trust score meaningfully reflects expert judgment, we analyzed both its discriminative ability and its probabilistic calibration. Discrimination was quantified using ROC curves that assess how well the AIF trustworthiness score separates samples that chemists judged as trustworthy (AIF or both) from those they considered untrustworthy (neither or lowest -Rwp). As shown in Figure S8, the area under the curve (AUC) ranges from 0.87 to 0.92 across the four chemists, with an overall AUC of 0.88. These values indicate strong alignment between AIF's internal confidence estimates and human trust assessments—samples with higher AIF trust scores are consistently more likely to be judged reliable by chemists.

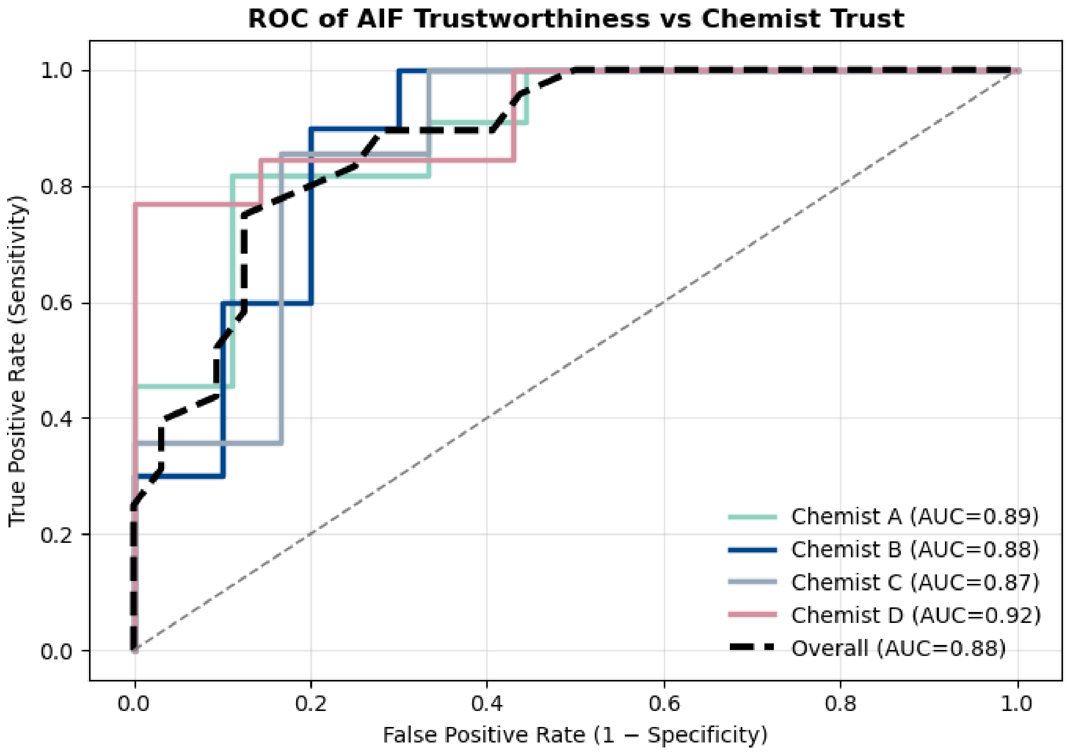

Figure S8: Receiver Operating Characteristic (ROC) curves evaluating how well the AIF trustworthiness score distinguishes between samples chemists considered trustworthy (AIF or both) and untrustworthy (neither or lowest-Rwp). Each curve corresponds to one chemist's evaluations, with the dashed black curve representing the aggregated overall ROC. AUC values between 0.87 and 0.92 indicate strong alignment between AIF's internal confidence and human trust judgments.

However, good discrimination does not guarantee good calibration. To assess whether AIF's trustworthiness score behaves like a meaningful probability, we computed Brier scores for each chemist and compared them against several baselines. The first baseline is a degenerate model that always predicts a trustworthiness of 1.0. A stronger baseline predicts a constant value equal to the mean of each chemist's empirical trust rate. This second baseline is calibrated but non-discriminative, so any useful trust metric should achieve a lower Brier score.

The Brier score for N samples is defined as:

$$\mathrm{BS} = \frac{1}{N}\sum_{t=1}^{N}(f_t - o_t)^2,$$

where f_t is the predicted trustworthiness for sample t and o_t is the observed outcome ($o_t = 1$ if the chemist trusted the interpretation, 0 otherwise). Lower values indicate better calibrated probabilities.

Figure S9 summarizes the results for both the multi-project evaluation and the Genome subset. In each case, the AIF trustworthiness score achieves lower (or comparable) Brier errors than constant-value baselines; the improvement is strongest in the multi-project benchmark and more modest in the Genome subset. This demonstrates that the trust metric is well calibrated: it assigns higher confidence to interpretations that chemists tend to trust, and lower confidence in ambiguous or contested cases.

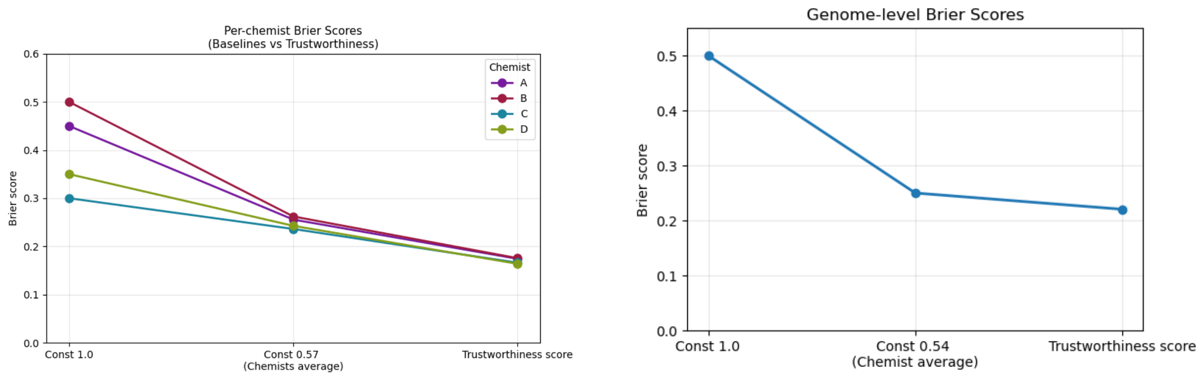

(a) Multi-project evaluation

(b) Genome dataset

Figure S9: Brier score analysis of AIF trustworthiness calibration. In both evaluations—the multi-project benchmark (left) and the precursor Genome subset (right)—AIF's trustworthiness metric outperforms constant-value baselines, demonstrating that the score is meaningfully calibrated. Lower values indicate better probabilistic accuracy.

13 Case studies

13.1 Chemically implausible interpretation rejected through chemical reasoning

Interp.	Phases	R_{wp}	Balance	Peak-matching score	LLM likel.	Trusted
I_1	CaC_2, CaV_2O_5	16.16	0.998	0.066	0.40	No
I_2	CaV_2O_5, $Ca_5V_3O_{13}$	16.27	0.992	0.151	0.75	Yes
I_3	CaC_2	18.05	0.375	0.031	0.10	No
I_4	$CaCO_3$	17.39	0.375	0.202	0.40	No

Table S6: Summary of key metrics for the four candidate interpretations in this case study.

13.2 Correct polymorph identified through chemical reasoning

A second example shows how the chemical knowledge layer can distinguish between polymorphs that give similar refinement quality. In this case, the target composition was a reduced calcium vanadate ternary oxide (CaV_2O_3), prepared via solid state synthesis from mixed precursors $CaCO_3$ (space group 167) and V_2O_3 (space group 167) at a 1:2 stoichiometric ratio of Ca:V, and heated at 500 °C for 12 h under flowing argon.

As shown in the first panel of Figure S10, three interpretations receive the highest posterior probabilities (81.9%, 78.4%, and 79.1%), all of which are composed of phases with the same compositions as the precursors, reflecting the fact that all three fit the diffraction data comparably well and have nearly identical balance scores. Because refinement-based metrics do not strongly differentiate them, these interpretations remain close in the probabilistic ranking. This indicates no ternary oxide was likely to have formed.

The phase-probability panel provides a clearer picture. The high temperature-stable V_2O_3 ($R\bar{3}c$, space group 167) polymorph accumulates the highest phase-level probability (67.4%), followed by the room temperature-stable $CaCO_3$($R\bar{3}c$, space group 167) precursor (57.0%). The high pressure metastable $CaCO_3$($P2_1/c$, space group 14) and low temperature V_2O_3($C2/c$, space group 15) polymorphs receive much lower probabilities (29.6% and 26.3%). This shift in probability mirrors the LLM phase-level likelihoods and boosts interpretations that contain the high temperature-stable, trigonal V_2O_3. Interpretation 1 therefore ends up slightly ahead in the full posterior. Table S7 summarizes the key metrics for all six candidate interpretations.

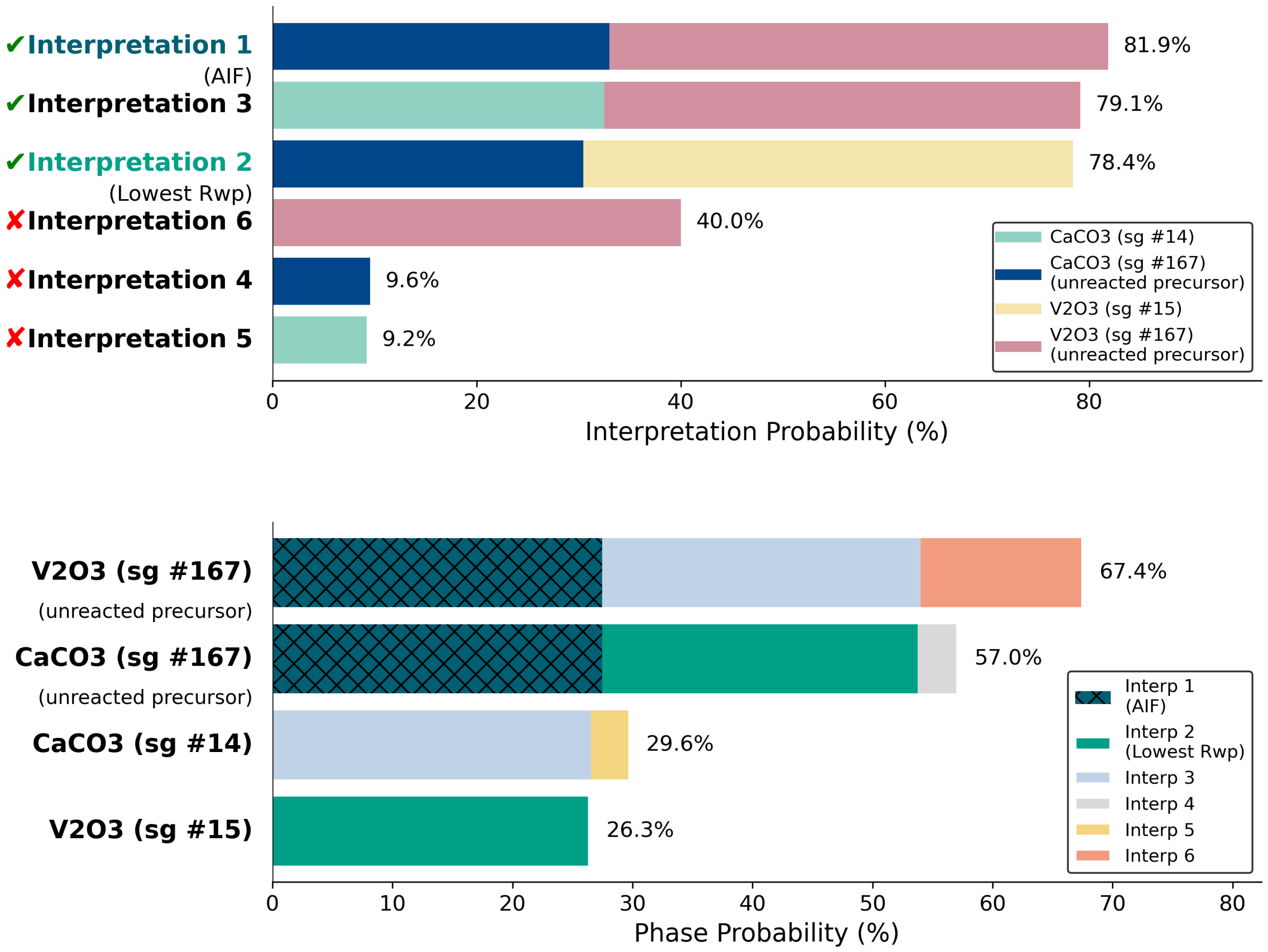

Figure S10: Probabilistic output of AIF for the CaV_2O_3 synthesis case study. The top panel shows posterior probabilities of candidate interpretations, with colored segments indicating the refined weight fractions of the constituent phases. The bottom panel shows phase-level posterior probabilities, with colors indicating the contribution of each interpretation.

Among the lowest-R_{wp} and AIF's interpretations the R_{wp} and balance scores are nearly indistinguishable, the key difference arises from the chemical-knowledge layer: the LLM prefers the high temperature-stable $R\bar{3}c$ V_2O_3(space group 167) polymorph over the low temperature $C2/c$ V_2O_3(space group 15) form. The LLM explanation is:

> *V2O3 is stable under reducing conditions, such as those provided by flowing argon. The space group 167 is consistent with the known structure of V2O3, making its formation likely. Space group 15 is less common for V2O3, suggesting a metastable or kinetically trapped phase. While possible, it is less likely than the stable polymorph.*

These differences shift the interpretation-level likelihoods, and AIF assigns higher probability to the interpretation that contains the high temperature-stable phase. We note that the LLM likelihood was generated using the precursor composition and was not aware of precursor space group. Following the analysis, it was confirmed that the polymorphs

selected by the LLM were indeed the same ones used as precursors, strengthening the confidence of the final AIF analysis.

Figure S11 shows how the four metrics contribute to the posterior probability for each interpretation. For the top three candidates (Interpretations 1–3), the R_{wp}, peak-matching score, and balance score are all similar, the LLM contribution is the main factor that distinguishes them.

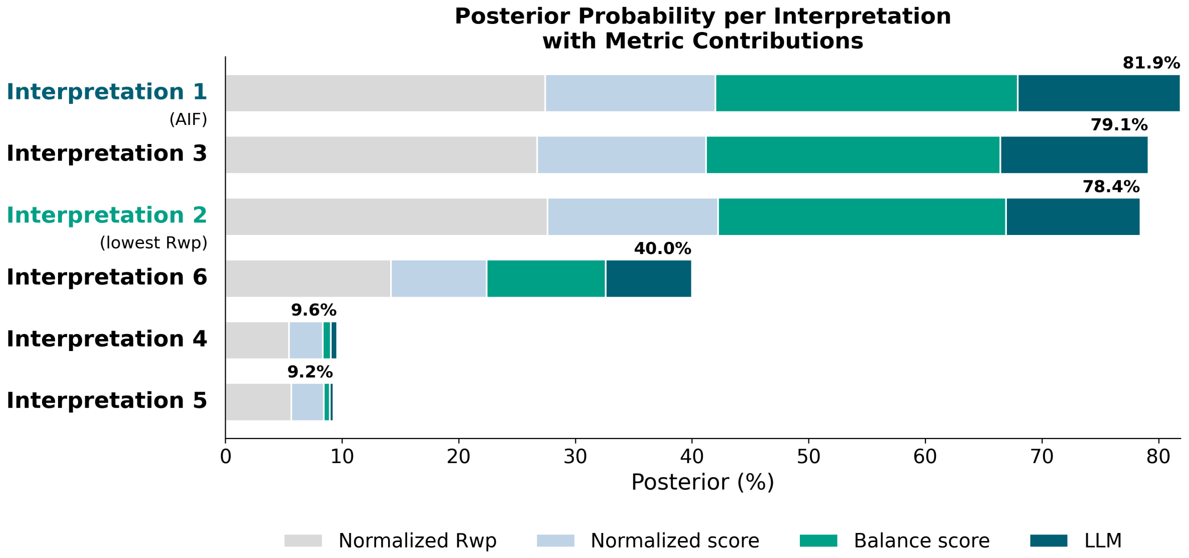

Figure S11: Metric-level contribution breakdown for the polymorph-selection case. Refinement-based metrics are similar across the top candidates, but the LLM contribution separates the chemically plausible interpretation containing the trigonal V_2O_3(space group 167) polymorph.

Interp.	Phases	R_{wp}	Balance	peak-matching	LLM likel.	Trusted
I_1	V_2O_3(167), $CaCO_3$(167)	6.18	0.996	0.882	0.75	Yes
I_2	V_2O_3(15), $CaCO_3$(167)	5.86	0.997	0.882	0.65	Yes
I_3	V_2O_3(167), $CaCO_3$(14)	7.01	0.994	0.886	0.70	Yes
I_4	$CaCO_3$(167)	30.75	0.185	0.256	0.20	No
I_5	$CaCO_3$(14)	27.30	0.185	0.270	0.15	No
I_6	V_2O_3(167)	21.73	0.593	0.518	0.60	No

Table S7: Key metrics for the candidate interpretations in the second case study. Interpretation 1 is the most chemically plausible and receives the highest AIF trust score.
